# A Smoking Cessation Mobile App for Persons Living With HIV: Preliminary Efficacy and Feasibility Study

**DOI:** 10.2196/28626

**Published:** 2022-08-18

**Authors:** Rebecca Schnall, Jianfang Liu, Gabriella Alvarez, Tiffany Porras, Sarah Ganzhorn, Samantha Boerner, Ming-Chun Huang, Paul Trujillo, Patricia Cioe

**Affiliations:** 1 Columbia University School of Nursing New York, NY United States; 2 Zucker School of Medicine Hofstra University Hempstead, NY United States; 3 Center for Psychedelic Medicine Department of Psychiatry NYU Langone Health New York, NY United States; 4 New York University Grossman School of Medicine New York, NY United States; 5 Bellevue Hospital Center New York, NY United States; 6 Case School of Engineering Case Western Reserve University Cleveland, OH United States; 7 Center for Alcohol and Addiction Studies Brown University Providence, RI United States

**Keywords:** HIV, mHealth, smoking cessation, intervention, smoking, persons with HIV, pilot, pilot test, mobile app, smartwatch

## Abstract

**Background:**

The prevalence of smoking in the United States general population has gradually declined to the lowest rate ever recorded; however, this has not been true for persons with HIV.

**Objective:**

We conducted a pilot test to assess the feasibility and efficacy of the Lumme Quit Smoking mobile app and smartwatch combination with sensing capabilities to improve smoking cessation in persons with HIV.

**Methods:**

A total of 40 participants were enrolled in the study and randomly assigned 1:1 to the control arm, which received an 8-week supply of nicotine replacement therapy, a 30-minute smoking cessation counseling session, and weekly check-in calls with study staff, or to the intervention arm, which additionally received the Lumme Quit Smoking app and smartwatch.

**Results:**

Of the 40 participants enrolled, 37 completed the follow-up study assessments and 16 used the app every day during the 56-day period. During the 6-month recruitment and enrollment period, 122 people were screened for eligibility, with 67.2% (82/122) deemed ineligible. Smoking criteria and incompatible tech were the major reasons for ineligibility. There was no difference in the proportion of 7-day point prevalence abstinence by study arm and no significant decrease in exhaled carbon monoxide for the intervention and control arms separately. However, the average exhaled carbon monoxide decreased over time when analyzing both arms together (*P*=.02).

**Conclusions:**

Results suggest excellent feasibility and acceptability of using a smoking sensor app among this smoking population. The knowledge gained from this research will enable the scientific community, clinicians, and community stakeholders to improve tobacco cessation outcomes for persons with HIV.

**Trial Registration:**

ClinicalTrials.gov NCT04808609; https://clinicaltrials.gov/ct2/show/NCT04808609

## Introduction

The prevalence of smoking in the general US population has gradually declined to 12.5% in 2020, the lowest rate ever recorded [1]. However, this has not been true for persons with HIV, who have disproportionately high smoking rates (34% to 47%) [2-4]. Across several studies of persons with HIV in New York City, Schnall et al [5] found that 47.1% of study participants were cigarette smokers, providing further evidence of the high prevalence of tobacco use in this population [6,7]. High rates of smoking have grave health implications for persons with HIV, placing them at increased risk for bacterial pneumonias [8], acute bronchitis and tuberculosis [9-16], early development of emphysema [17-22], and lung and cervical cancers at a younger age than the general population [23-28]. These disparities are partly due to a higher prevalence of tobacco use in persons with HIV than the general population and partly attributable to exacerbated comorbidities such as cardiovascular disease, lung cancer, and diabetes in persons with HIV who smoke compared to smokers without HIV [29-31]. For persons with HIV, apart from achieving and maintaining a suppressed viral load, tobacco cessation is the most important health behavior they can undertake to maximize both quality of life and life expectancy [32]. Indeed, persons with HIV who quit smoking upon entering HIV care gain more than 5 years of life expectancy as compared to those who enter HIV care and continue smoking [33,34]. Consequently, persons with HIV experience substantial tobacco-related morbidity and mortality.

Given the high prevalence of cigarette smoking among persons with HIV and the benefit of smoking cessation, there is an urgent need to intervene to reduce tobacco use rates. However, the currently available evidence for improving tobacco cessation among persons with HIV is inadequate. Few tobacco cessation interventions have been tested among persons with HIV, and of those that have, there is very low quality evidence that they were effective in the short term and moderate quality evidence indicating similar outcomes to controls in the long term [35-39].

In recent years, the use of mobile health (mHealth) technologies for tobacco cessation, such as Text2Quit [40] and SmokeFree Text from the National Cancer Institute [41], has gained popularity. While these programs have demonstrated positive effects in some populations [42-45], their functionality is limited because text messaging is unable to automatically capture instances when assistance is required to resist cravings or when smoking relapse occurs. To address this need, our study sought to use more advanced technology, comprising a mobile app and smartwatch, that functioned as a sensor to integrate behavioral assessment and a just-in-time cessation intervention for smokers. The app and watch sensor provided smoking relapse prevention to supplement the cessation intervention of traditional nicotine replacement therapy (NRT). Watch sensor data, in conjunction with the app, functioned to prevent smoking relapse immediately following the initiation of NRT (quit date). The purpose of this pilot study was to determine the feasibility and acceptability of the Lumme Quit Smoking app for improving tobacco cessation outcomes in persons with HIV who smoke.

## Methods

### Study Design

The study was conducted with 40 persons with HIV who smoke who were randomized to 2 study arms. Participants were randomly assigned to receive the Lumme Quit Smoking app (active) or a control condition (standard smoking cessation counseling session). Participants had in-person study visits at baseline and 12 weeks following their baseline visit. The primary objectives of the study were to assess the feasibility and acceptability of the Lumme Quit Smoking app; the study was not powered to assess efficacy. A secondary goal was to establish the effect size needed to conduct a fully powered intervention trial.

### Recruitment

Study participants were recruited by posting flyers at a local HIV clinic and from an existing database of persons living with HIV who had participated in earlier studies and agreed to be recontacted for future studies. Study enrollment took place from September 28, 2020, to April 19, 2021.

Inclusion criteria were (1) persons with HIV, (2) aged 18 years and older, (3) own an Android smartphone, (4) understand and read English, (5) not pregnant or breastfeeding, (6) have permanent contact information, (7) smoke 5 or more cigarettes per day for the past 30 days [46], (8) interested in quitting smoking within 30 days, and (9) have an exhaled carbon monoxide (eCO) level of more than 5 parts per million (ppm) at baseline.

Exclusion criteria were (1) self-report having HIV-negative or unknown status; (2) pregnant, breastfeeding, or planning to become pregnant during the study period; (3) planning to move within 3 months of enrollment; (4) positive history of a medical condition that precludes use of the nicotine patch; (5) current use of NRT or other smoking cessation medications (eg, Chantix or Zyban); (6) current enrollment in another smoking cessation program; or (7) eCO of 5 ppm or less at baseline.

Biochemical verification of abstinence appears to be increasingly important, especially in clinical trials, and increases scientific rigor, as both social norms relating to smoking behavior and an increasing number of personal factors (eg, age, pregnancy, hospitalization status, and socioeconomic status) are related to misreporting of smoking behavior. eCO is a useful assessment tool for measuring recent smoking abstinence [47].

### Ethics Approval

The Columbia University institutional review board approved this study before commencement of study activities, and the study was registered with ClinicalTrials.gov [NCT04808609].

### Procedures

Potential study participants provided verbal informed consent to complete a phone screening to assess eligibility. Eligible participants were then invited to attend an in-person baseline visit at the Columbia University School of Nursing. Upon arrival to the study site, scheduled participants were asked to provide a breath sample to determine eCO level and confirm study eligibility. Breath samples were analyzed for eCO levels (in ppm) using a breathalyzer (Micro Basic Smokerlyzer, Bedfont Scientific Ltd). Those identified as ineligible were given a Metrocard to thank them for their time. Those identified as initially eligible were asked to complete a written informed consent form. Study staff guided participants through the form and answered any questions.

After providing written consent, participants completed a baseline questionnaire that included demographic characteristics, tobacco use history, illicit substance use history, alcohol use history, psychosocial characteristics, and pharmacotherapy use (Multimedia Appendix 1). Survey instruments were collected via Qualtrics. Following completion of the baseline study assessments, study participants were randomized (1:1) to the Lumme Quit Smoking app arm or the control arm.

### Randomization

We did not stratify the sample by any demographic characteristic (ie, age, sex, gender, race, or ethnicity) since this was a pilot study with a small sample. We concealed randomization status from staff and participants until after completion of the baseline assessment to minimize bias. The study statistician who performed the data analysis was blinded to the treatment groups.

Participants in both arms received a smoking cessation counseling session and were provided with NRT in the form of the NicoDerm CQ Patch (GSK Group of Companies) under the supervision of a nurse. Each participant received an 8-week supply of NRT, which was enough to follow the 3-step program. However, they were encouraged not to begin NRT until 2 weeks after the baseline visit. Participants in both study arms set a quit date for 2 weeks after the baseline visit. Dosing of the NRT was based on standard prescribing guidelines. Participants in both arms also received a 30-minute smoking cessation counseling session and weekly check-in calls from the study staff.

**Figure 1:**
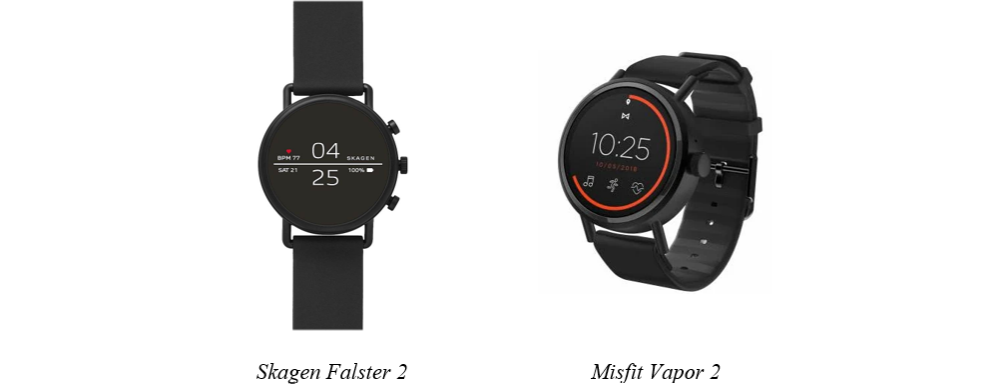
Sample picture of smartwatches used by study participants.

**Figure 2:**
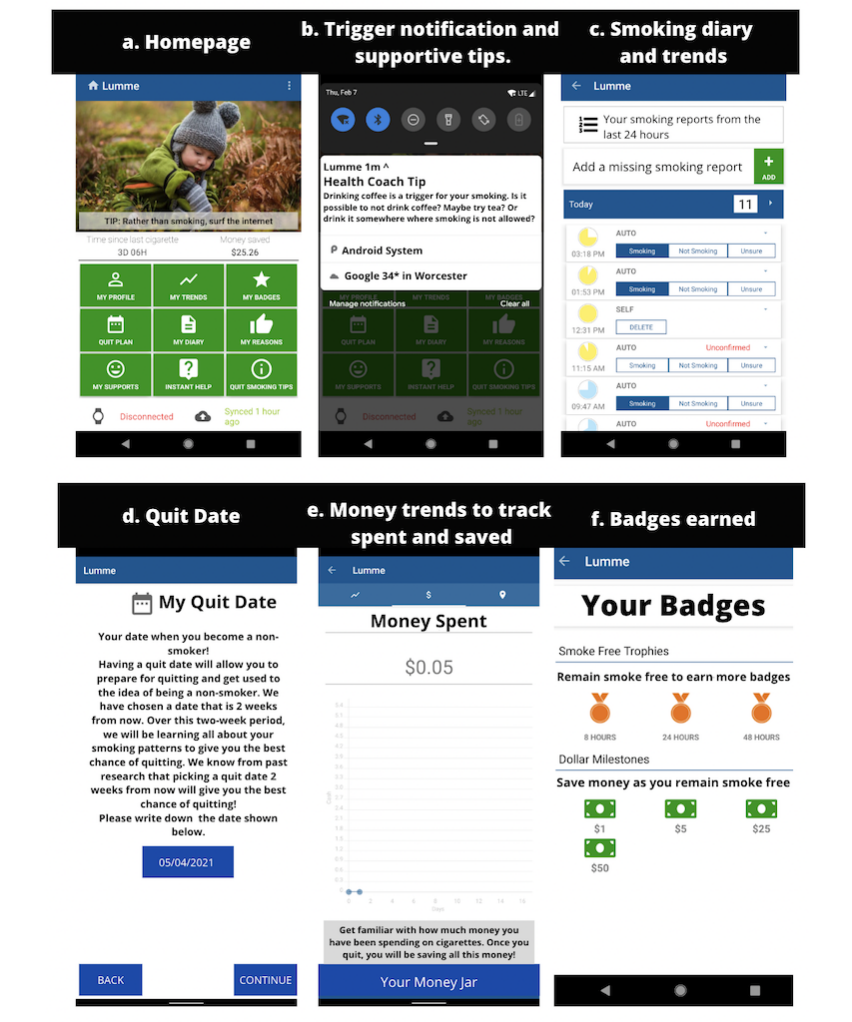
Screenshots of the Lumme Quit Smoking app.

Participants in the intervention arm received a Vapor 2 (Misfit) or Falster 2 (Skagen Denmark) smartwatch (Figure 1). Both smartwatches are compatible with the Lumme Quit Smoking app. The Lumme Quit Smoking app was paired with the smartwatch so that smoking was detected by the smartwatch and the information sent directly to the Lumme Quit Smoking app. The smartwatch detected hand movements indicating a study participant was picking up a cigarette to smoke or a user manually entered a smoking session. The Lumme Quit Smoking app was then able to predict cravings, target users with notifications to prevent individuals from smoking, refine the notifications for each user, and display their change in smoking behavior and money saved in a smoking diary (Figure 2). Users were also able to see their quit plan with their assigned quit date for 2 weeks after baseline, along with smoking trends, supportive tips, and badges earned from the amount of money saved.

Intervention arm participants created an account with the Lumme Quit Smoking app, which passively collected smoking data, such as when the user smoked (time of day, day of week, before/after eating, after waking up/before going to sleep, before driving to/from work, while driving), where the user smoked, and who was nearby when the user smoked (based on repeated detection of the same Bluetooth static address; Lumme does not collect identities), during the first 2 weeks of the study period. At the end of the baseline visit, all enrolled participants were compensated $40 for their participation in the form of a preloaded debit card. On the day before their quit date, study staff under the supervision of a nurse called participants in both arms to remind them of their quit date and to start using their NRT patches on the morning of their quit date. Additionally, participants in both the control and intervention arm received weekly check-in calls from study staff members. Staff conducted these calls to monitor adherence to NRT, to provide technology assistance to those in the intervention arm who might be experiencing difficulties with the smartwatch or app, and to encourage participant engagement in the study.

### Study Measures

To determine feasibility, we examined retentions rates, compliance rates, dosing, eligibility criteria, recruitment and enrollment rates, missing data, and study measures. Retention rates were measured as the number of enrollees who remained in the study; 80% retention was the threshold for feasibility. Compliance rates were calculated as the number of days participants used the Lumme Quit Smoking app divided by the 56 days of the study intervention program with at least 80% of participants having completed at least 75% of the intervention content set as the threshold. Dose was measured as the number of app sessions completed by the participants. Eligibility criteria were assessed to determine if the number of individuals who screened out of the pilot study was too high, indicating criteria that were too stringent. Recruitment and enrollment pace were assessed by dividing the total number of enrolled participants by the number of months in the recruitment period. The study measures and missing data were evaluated to determine if the survey questions needed adjustment by assessing the extent and patterns of missing data and the length of time needed to complete the study measures.

Primary outcome 7-day point prevalence abstinence was measured by self-report and biochemically verified via eCO at 12 weeks. Baseline nicotine dependence was measured using the Fagerström Test for Nicotine Dependence [48]. Baseline tobacco use was measured through self-reported number of cigarettes smoked per day. Baseline alcohol use was measured using the CAGE (cut, annoyed, guilty, and eye) Alcohol Abuse Screening Tool [49]. Depression was measured using the Center for Epidemiologic Studies–Depression Scale (CES-D) at baseline and 12 weeks [50].

At the 12-week follow-up visit, semistructured in-depth interviews were conducted with study participants. No additional compensation was provided for participation in the interviews. These interviews aim to fill gaps in the literature by taking a qualitative approach to understanding the acceptability of the Lumme app as a smoking cessation tool in the intervention arm and learn more about the perceptions of all participants on the recruitment and enrollment process. Both groups answered the following questions: How would you modify the outreach and recruitment process to improve it or make it easier? How comfortable were you with the recruitment, screening, and enrollment process? How can we improve the current screening process? The intervention group completed a questionnaire (Multimedia Appendix 2-5) that included the following questions, among others: Please describe your general perceptions and expectations of the app. How often did you use the app? How helpful was the app for tobacco cessation?

### Data Analysis

Descriptive statistics were used to detail the study sample by condition and as a total sample. Descriptive statistic *P* values were calculated by *t* test, chi-square test, or Fisher exact test. Point estimates and corresponding confidence intervals were calculated for each measure by arm. Because of our small sample sizes, we used Clopper-Pearson exact confidence intervals based on the binomial distribution and reported effect sizes (using relative risk or Cohen *d* depending on the distribution of the outcome measures) as well as *P* value (calculated by chi-square test for binary outcomes and independent samples *t* test for continuous outcomes) to examine differences in primary outcome (7-day point prevalence abstinence) and secondary outcomes (eCO decrease from baseline to 12-week follow-up, self-reported number of cigarettes smoked per day, and CES-D and CAGE scores) by study arms. The 7-day point prevalence abstinence was calculated as the proportion of participants who reported no smoking/tobacco use in the 7 days prior to their 12-week follow-up visit, biochemically verified by eCO collected at 12 weeks. Participants with eCO levels less than 6 ppm at 12 weeks were classified as abstinent while participants with eCO levels 6 ppm or more at 12 weeks were classified as not abstinent. Participants lost to follow-up were included in the analyses as smokers. We also assessed the time trends of eCO by each study arm and in both arms using paired *t* tests (used to calculate effect size and *P* value) because a total sample size of 37 is sufficient to detect a medium effect size when assessing within-subject effect, the time trend effect. All analyses were performed in SAS (version 9.4, SAS Institute Inc).

All in-depth interviews were transcribed verbatim and then coded using the Fit Between Individuals, Task, and Technology (FITT) framework along 3 dimensions: task-technology fit, individual-technology fit, and individual-task fit [51]. The FITT framework has been successfully used to determine the usability of mHealth technology in prior studies, proving to be a useful framework to guide this analysis [52,53].

## Results

### Demographic Characteristics

The demographic characteristics of participants are described in Table 1. There was no significant difference in study characteristics between study conditions. The mean age of study participants was 54 years, 50% (20/40) of participants identified as male, 78% (31/40) identified as Black/African American, and 23% (9/40) identified as Hispanic/Latinx. Participants had a mean CES-D score of 15.65 (SD 11.1). CAGE scores indicated that 22% (8/37) of participants had clinical indications of an alcohol use problem.

**Table 1:** Participant characteristics at baseline (n=40).

Characteristics			Total (n=40)^a^		Intervention (n=20)		Control (n=20)		*P* value
Age (years), mean (SD)			53.7 (9.2)		53.4 (10.2)		54.0 (8.5)		.84
**Gender identity, n (%)**			—^b^		—		—		>.99
	Male	20 (50)		10 (50)		10 (50)		—	
	Female	18 (45)		9 (45)		9 (45)		—	
	Transgender female	1 (3)		0 (0)		1 (5)		—	
	Genderqueer	1 (3)		1 (5)		0 (0)		—	
**Sex at birth, n (%)**			—		—		—		>.99
	Male	22 (55)		11 (55)		11 (55)		—	
	Female	18 (45)		9 (45)		9 (45)		—	
**Race, n (%)**			—		—		—		.06
	Black/African American	31 (78)		13 (65)		18 (90)		—	
	White	1 (3)		0 (0)		1 (5)		—	
	Multiracial	1 (3)		1 (5)		0 (0)		—	
	Unknown	7 (18)		6 (30)		1 (5)		—	
**Hispanic/Latinx ethnicity, n (%)**			—		—		—		.13
	Yes	9 (23)		7 (35)		2 (10)		—	
	No	31 (78)		13 (65)		18 (90)		—	
**Education, n (%)**			—		—		—		.11
	None	2 (5)		0 (0)		2 (10)		—	
	Some high school, no diploma	12 (30)		7 (35)		5 (25)		—	
	High school diploma or equivalent	14 (35)		8 (40)		6 (30)		—	
	Some college	8 (20)		2 (10)		6 (30)		—	
	Associate degree or technical degree	3 (8)		3 (15)		0 (0)		—	
	Bachelor/college degree	1 (3)		0 (0)		1 (5)		—	
**Employment, n (%)**			—		—		—		.63
	Working full-time	1 (3)		1 (5)		0 (0)		—	
	Working part-time	4 (10)		1 (5)		3 (15)		—	
	Unemployed	13 (33)		6 (30)		7 (35)		—	
	Retired	4 (10)		3 (15)		1 (5)		—	
	Disabled	15 (38)		7 (35)		8 (40)		—	
	Retired and disabled	1 (3)		1 (5)		0 (0)		—	
	Unemployed and disabled	1 (3)		0 (0)		1 (5)		—	
	Working part-time and retired	1 (3)		1 (5)		0 (0)		—	
**Annual income (US $), n (%)**			—		—		—		.88
	<10,000	17 (43)		7 (35)		10 (50)		—	
	10,000-19,999	12 (30)		7 (35)		5 (25)		—	
	20,000-39,999	2 (5)		1 (5)		1 (5)		—	
	40,000-59,999	1 (3)		1 (5)		0 (0)		—	
	Don’t know	8 (20)		4 (20)		4 (20)		—	
**Health insurance, n (%)**			—		—		—		.16
	Health exchange	1 (3)		1 (5)		0 (0)		—	
	Medicaid/Medicare	35 (88)		19 (95)		16 (80)		—	
	AIDS Drug Assistance Program	1 (3)		0 (0)		1 (5)		—	
	Uninsured	3 (8)		0 (0)		3 (15)		—	
**Quit attempt in past year, n (%)**			—		—		—		.75
	Yes	19 (48)		9 (45)		10 (50)		—	
	No	21 (53)		11 (55)		10 (50)		—	
**Cigarette type used, n (%)**			—		—		—		.61
	Menthol	36 (90)		19 (95)		17 (85)		—	
	Nonmenthol	4 (10)		1 (5)		3 (15)		—	
**Usual cigarette brand^c^, n (%)**			—		—		—		.61
	Marlboro	3 (8)		1 (5)		2 (11)		—	
	Newport	36 (92)		19 (95)		17 (90)		—	
**CAGE^d^ Substance Abuse Screening Tool^e^, n (%)**			—		—		—		>.99
	Clinically significant alcohol problems indicated	8 (22)		4 (21)		4 (22)		—	
	Clinically significant alcohol problems not indicated	29 (78)		15 (79)		14 (78)		—	
CES-D^f^ score, mean (SD)			15.7 (11.1)		16.6 (13.1)		14.8 (8.9)		.61
eCO^g^ (ppm^h^), mean (SD)			13.8 (6.4)		14.5 (6.5)		13.1 (6.3)		.48
Number of cigarettes smoked daily in past 30 days, mean (SD)			10.2 (5.3)		11.1 (6.7)		9.3 (3.3)		.31
Number of years smoking, mean (SD)			33.4 (12.1)		34.2 (10.7)		32.7 (13.6)		.70
Fagerström Test for Nicotine Dependence, mean (SD)			6.4 (2.0)		6.7 (1.7)		6.2 (2.3)		.48

^a^Column percentages may not sum to 100% due to rounding.

^b^Not applicable.

^c^n=1 missing.

^d^CAGE: cut, annoyed, guilty, and eye.

^e^n=3 missing.

^f^CES-D: Center for Epidemiologic Studies–Depression Scale.

^g^eCO: exhaled carbon monoxide.

^h^ppm: parts per million.

### Smoking Characteristics

Participants had a mean eCO at baseline of 13.8 (SD 6.4) and reported smoking an average of 11.5 (SD 4.7) cigarettes daily in the past month. Participants reported smoking for a mean of 33 (SD 12) years. Menthol cigarettes were used by 90% (36/40) of participants and 92% (36/39) reported that Newport was their usual cigarette brand. Per the Fagerström Test for Nicotine Dependence, participants had a mean nicotine dependence level of 6.4 (SD 2.0). Nearly half of participants (19/40, 48%) reported trying to quit smoking in the past year. There were no significant differences in smoking characteristics by study arm.

### Feasibility and Retention Rates

**Figure 3:**
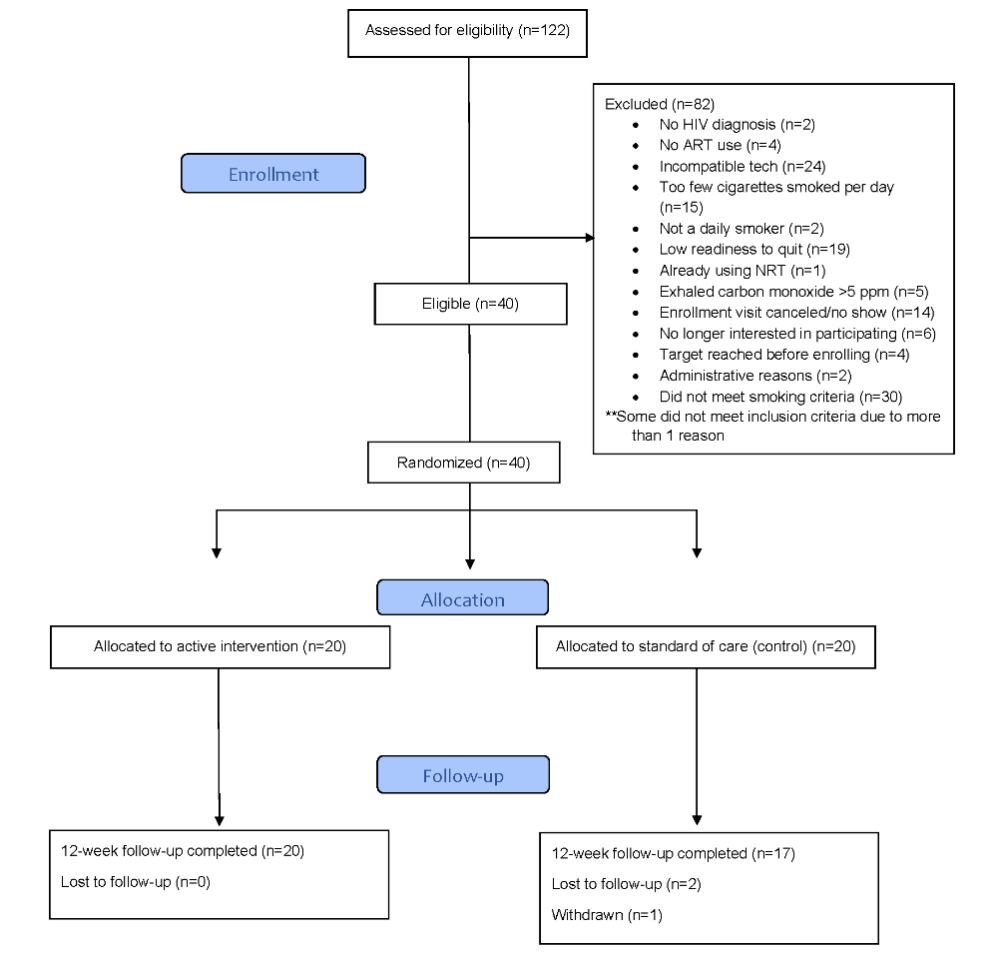
Smoking cessation pilot CONSORT (Consolidated Standards of Reporting Trials) flowchart. ART: antiretroviral therapy; NRT: nicotine replacement therapy.

Of the 40 participants who enrolled in the study, 37 returned to complete the follow-up study assessment at 12 weeks. Of the 3 participants who did not finish the study, 1 was withdrawn and 2 were lost to follow-up. The 3 participants who failed to complete the study were all assigned to the control arm (Figure 3). Dose was operationalized as the number of app sessions completed by the participant; 80% (16/20) of participants used the app every day during the 56-day period. Of the 16 participants, 14 used the Lumme Quit Smoking app for at least 2 weeks past the official program end date.

### Eligibility Criteria

During the recruitment and enrollment period, 122 people were screened for eligibility with 67.2% (82/122) screening ineligible. Smoking criteria and incompatible tech were the most frequently occurring reasons for ineligibility. During the study’s 6-month recruitment and enrollment period, approximately 6 participants were enrolled per month.

### Study Measures and Missing Data

All 40 enrolled participants completed the baseline survey, and 37 participants completed the follow-up survey at 12 weeks. Among the 77 completed survey responses, missing data were evenly distributed across study arms. On average, study participants completed the baseline survey in 79 (SD 45) minutes and completed the follow-up survey in 30 (SD 16) minutes.

### Acceptability

Table 2 displays descriptive statistics and associated 95% confidence intervals for primary, secondary, and other outcome measures.

The relative risk of 7-day prevalence abstinence was 1.27 between the intervention and control groups, indicating that the 7-day abstinence prevalence was 27% higher in the intervention group than in the control group; the effect size of eCO decrease was small (Cohen *d*=0.21); for other secondary outcomes, the effect sizes were less than small (Cohen *d*<0.20). There was no significant difference in decrease in eCO for the control arm or intervention arm. Average eCO decreased significantly over time (baseline 13.8 [SD 6.4], 12-week follow-up 11.4 [SD 8.2]; *P*=.02) when analyzing both arms together (Table 3).

**Table 2:** Differences in outcome measures by study arm.

Measure	Control (n=20)^a^			Intervention (n=20)			Total (n=40)			Effect size (95% CI)		*P* value
	Value	95% CI	Value		95% CI	Value		95% CI				
7-day point prevalence abstinence verified via eCO^b^ at 12 weeks, n (%)	2 (12)	1.5 to 36.4	3 (15)		3.2 to 37.9	5 (14)		4.5 to 28.8	1.27 (0.24 to 6.76)		.77	
Self-report 7-day point prevalence abstinence at 12 weeks, n (%)	4 (24)	6.8 to 49.9	6 (30)		11.9 to 54.3	10 (27)		13.8 to 44.1	1.27 (0.43 to 3.78)		.66	
eCO ppm^c^ decrease from baseline to 12 weeks, mean	17 (2.12)	–0.9 to 5.14	20 (3.35)		–0.2 to 6.92	37 (2.78)		0.69 to 4.88	0.21 (–0.44 to 0.86)		.59	
CES-D^d^ score, mean	20 (14.8)	10.8 to 18.7	20 (16.6)		10.8 to 22.3	40 (15.7)		12.2 to 19.1	0.16 (–0.46 to 0.78)		.61	
Number of cigarettes smoked per day reported at baseline, mean	20 (11)	9.3 to 12.7	20 (11.9)		9.5 to 14.3	40 (11.5)		10 to 12.9	0.19 (–0.42 to 0.80)		.55	
CAGE^e^ Substance Abuse Screening Tool score at baseline, mean	18 (0.9)	0.3 to 1.5	19 (0.9)		0.2 to 1.6	37 (0.9)		0.5 to 1.3	0.004 (–0.61 to 0.62)		.99	

^a^n=3 missing in control group at follow-up.

^b^eCO: exhaled carbon monoxide.

^c^ppm: parts per million.

^d^CES-D: Center for Epidemiologic Studies–Depression Scale.

^e^CAGE: cut, annoyed, guilty, and eye.

**Table 3:** Change in exhaled carbon monoxide in parts per million from baseline to 12 weeks by study arm.

Arm	Baseline eCO^a^		eCO at 12 weeks			Effect sizes in change (95% CI)	*P* value
	Participants, n	Mean (SD)	Participants, n	Mean (SD)			
Control	20	13.1 (6.3)	17	11.6 (7.4)	0.36 (–0.14 to 0.85)		.16
Intervention	20	14.5 (6.5)	20	11.2 (9.0)	0.44 (–0.03 to 0.89)		.06
Both	40	13.8 (6.4)	37	11.4 (8.2)	0.41 (0.07 to 0.74)		.02

^a^eCO: exhaled carbon monoxide.

### NRT Use

At the follow-up study visit, participants self-reported their NRT use. A total of 46% (17/37) of all study participants reported daily NRT use, 45% (9/20) in the intervention arm reported daily NRT use, and 47% (8/17) in the control arm reported daily NRT use. There was no significant difference in NRT use between participants in the study arms.

### In-depth Interviews

Findings from the in-depth interviews are organized according to the FITT framework, which provided for a better understanding of the factors contributing to the engagement and perceived usefulness of the intervention. Sample quotes that are exemplars of each of the constructs of the framework are detailed in Table 4.

**Table 4:** Exemplars of findings from the in-depth interviews organized by the Fit Between Individuals, Task, and Technology framework.

FITT^a^ framework construct	Sample quotes from in-depth interview
Task-technology fit	“And it does really help you. It tells you when your day is over. It goes, ‘You have such-and-such more days before you quit.’ That was a good reminder...” “I looked at it a couple times a day. In the beginning when I was smoking, I looked at it almost every time I smoked just to see if it recorded. But after I stopped smoking, I would look at it still...” “...this watch is going to monitor my movement of my hand... I’m not going to be smoking all the time and moving my hand... I already knew that... that I would have to check in and be honest. Honesty was one of my biggest, like I expected to be honest. And that’s what I’ve been...”
Individual-technology fit	“The tips were very useful because everything it says in the tips is true…pros and cons…which is another way you can sit there and say is smoking good or bad. Having the support system on there was cool... anytime I wanted to I could contact the support system anytime I needed to speak to somebody...” “[I used the watch] everywhere. If I was in the bathroom; if I was making a delivery if I was at work. It didn’t matter where it was... I was able to text through the watch... I find that very cool, too.” “I think this whole experience was something good for me... I’ve always wanted to stop smoking... I’ve slowed down enough to maybe be able to stop. And it gave me the encouragement, the power and the push to do it in a way that’s not hard...” [51]
Individual-task fit	“It’s extremely helpful, it definitely helped me cut back. I’m more confident with the app and like I said, it’s like a little coach with you all the time, 24/7....” “...[the app] did make things very clear, as far as really specifying your habits... In that respect, it’s been good, because it made you conscious of how much you’re smoking, even though you do it normally without the app and you just smoke, and you don’t think about it...” “[The app was] 100% helpful for an individual that’s really focused on not smoking, on quitting, being focused on quitting and wanting to... I mean that was amazing... to have a watch on and when you’re going like this or you know... they always give you the option to say, well, no, I wasn’t smoking at that time; I was eating or something like that... the individual has to be honest, with themselves...”

^a^FITT: Fit Between Individuals, Task, and Technology.

## Discussion

### Principal Findings

This study focused on testing a multicomponent intervention, Lumme Quit Smoking app with nicotine replacement therapy (NRT) and smoking cessation counseling to improve smoking cessation in persons living with HIV. This is especially important given that tobacco use causes increased morbidity and mortality in persons with HIV, and tobacco-related harm is substantially higher in persons with HIV than in smokers in the general population. There were several innovative components of this intervention. The algorithm for detecting the smoking arm movement can be used to interpret data from a smartwatch [54]. The Lumme Quit Smoking app uses a validated sensor to detect participants’ smoking behaviors in real time. This intervention combined biometric data with behavioral interventions specific to smoking behavior, an advancement and innovation not previously tested in persons with HIV. Findings from this study suggest that the intervention was both feasible and acceptable in this population. Participants wore the sensor and used the app. Retention in the study was very good. The high completion rate and continued use of the app for weeks beyond the program end indicates that the dose was not burdensome and could be extended for future smoking cessation studies. Notably, the loss to follow-up and withdrawal were all among control group participants. Therefore, consideration of whether this loss to follow-up is associated with treatment group will need to be considered in the next trial, although it is noted that retention was overall very good, and numbers of participants who dropped out of the study are small.

This pilot study was underpowered to detect statistically significant differences between conditions on efficacy outcomes. There were only 2 participants who quit the smoking in the control group and 3 in the intervention group, verified through biochemical testing. Based on the self-reported quit rates, there were 4 (control) versus 6 (intervention) participants who quit smoking. Nonetheless, these findings allowed us to estimate that the intervention was able to improve the prevalence of 7-day abstinence by 27% in the study sample, and there was a decrease in the eCO over time with an almost medium effect size (Cohen *d*=0.44) in the intervention group.

The ability for a mobile app to deliver content that would burden participants and require staff and clinician time and resources is an innovative approach to the delivery of a tobacco cessation intervention. Importantly, mHealth is a feasible platform for delivering this intervention since there is extremely high mobile phone penetration in the United States [55], especially among racial/ethnic minority groups [56]. mHealth technology can be used for achieving health equity in vulnerable groups because it is a widely available and relatively inexpensive tool for health behavior change [57] and can be adapted to meet the needs of its end users [58-61]. Our work has shown that even the lowest income and most health disparate persons own and use smartphones [62]. Therefore, the Lumme Quit Smoking app is timely, relevant, scalable, and likely to improve health outcomes in persons with HIV who smoke. Even when successful, however, there are barriers to widespread adoption and successful scale-up of tobacco interventions [63,64]. While leveraging accessible mHealth technologies is a strength, our study team acknowledges the target population may not have access to smartwatches outside of the research study. Nonetheless, if efficacious, the cost of the smartwatch is half of the cost of purchasing cigarettes for 1 month in New York City, estimated at $320 per month [65] and even more minimal in comparison to the annual cost ($170 billion) for direct medical care for persons who smoke in the United States [66].

### Limitations

There were several important limitations of this study. First, we relied on self-report of NRT use among study participants, and while we tracked our distribution of NRT to study participants, we were limited in our ability to validate whether participants used the NRT. We carefully considered our control condition and ultimately the robustness of the control condition (smoking cessation counseling session and NRT administration) may have underestimated the effect of the Lumme Quit Smoking app.

### Conclusion

Notably, smoking cessation efforts have largely been aimed at the general population. Consequently, it is not clear whether they are suitable or effective for cohorts with population-specific concerns and clinical issues such as persons with HIV [11,22,67,68]. While the Lumme Quit Smoking app is undeniably an innovation in combining sensors, real-time feedback, and behavioral interventions, it was developed for the general population of smokers and was not in any way targeted or tailored for the needs of persons with HIV. This may have contributed to the findings that this intervention was not significantly more efficacious at improving smoking cessation than a standard of care control condition. Future research should focus on identifying the behavioral factors specific to persons with HIV who smoke and developing an intervention to meet their needs. Additionally, the counseling component of the intervention may need to be tailored to persons with HIV. A recent study found that persons with HIV did not understand the relationship between HIV and smoking and described wanting more information about the health effects of smoking for persons with HIV [69].

## Appendix

Multimedia Appendix 1 Fagerstrom Test for Nicotine Dependence.

Multimedia Appendix 2 Smoking cessation pilot interview guide: control.

Multimedia Appendix 3 Smoking cessation pilot interview guide: intervention.

Multimedia Appendix 4 Smoking cessation pilot: baseline survey.

Multimedia Appendix 5 Smoking cessation pilot: follow-up survey.

## Abbreviations

CAGE: cut, annoyed, guilty, and eye
CES-D: Center for Epidemiologic Studies–Depression Scale
eCO: exhaled carbon monoxide
FITT: Fit Between Individuals, Task, and Technology
mHealth: mobile health technologies
NRT: nicotine replacement therapy
ppm: parts per million
