# Supplementary material for: A Smoking Cessation Mobile App for Persons Living With HIV: Preliminary Efficacy and Feasibility Study
Source: JMIR Form Res. 2022 Aug 18;6(8):e28626. doi: 10.2196/28626 (PMC9437787; doi:10.2196/28626)
Supplement: Multimedia Appendix 1 [file formative_v6i8e28626_app1.pdf]

Fagerström Test for Nicotine Dependence

Agency Name: ________________________ **Site Name:** ________________________

ID #: ________________________ **Date:** __ __ / __ __ / __ __ __ __

Please read each question below. For each question check the answer choice which best describes your responses.

1. How soon after you wake up do you smoke your first cigarette?

- Within 5 minutes ❑3
- Within 6 - 30 minutes ❑2
- Within 31 - 60 minutes ❑1
- After 60 minutes ❑0

2. Do you find it difficult to refrain from smoking in places where it is forbidden, e.g., in church, at the library, at the movies, etc.?

- No ❑0
- Yes ❑1

3. Which cigarette would you hate most to give up?

- The first one in the morning ❑1
- All others ❑2

4. How many cigarettes per day do you smoke?

- 10 or less ❑1
- 11 - 20 ❑2
- 21 - 30 ❑3
- 31 or more ❑4

5. Do you smoke more frequently during the first hours after waking than during the rest of the day?

- No ❑0
- Yes ❑1

6. Do you smoke if you are so ill that you are in bed most of the day?

- No ❑0
- Yes ❑1

Reference: Heatherton TF; Kozlowski LT; Frecker RC; Fagerstrom KO. The Fagerstrom Test for Nicotine Dependence : a revision of the Fagerstrom Tolerance Questionnaire. Br J Addict 1991;86:1119-1127.
