# Supplementary material for: A Smoking Cessation Mobile App for Persons Living With HIV: Preliminary Efficacy and Feasibility Study
Source: JMIR Form Res. 2022 Aug 18;6(8):e28626. doi: 10.2196/28626 (PMC9437787; doi:10.2196/28626)
Supplement: Multimedia Appendix 2 [file formative_v6i8e28626_app2.pdf]

Protocol: AAAS6990
Study: Smoking Cessation Pilot

Interview Start

Thank you for taking the time to participate in this interview today. My name is __________, I will be interviewing you today.

You were invited because you participated in the Smoking Cessation study. The purpose of today's talk is to gather information about your experience as well as your perception on participating in the study.

There are no right or wrong answers to the questions I am about to ask. Please feel free to share your thoughts.

Before we begin, I want to mention some ground rules:

We are recording the session because we don't want to miss any of your comments as all your comments are valuable to us. We will remove any references to specific people from the written notes and transcripts.

You are welcome to use a pseudonym or fictional name, but it's not required. You can use your own name. Friendly reminder that personal information that identifies you will be removed later during the analysis.

I will be taking notes to help myself remember what you said.

If you don't mind, please turn off/silence your cell phone so we can begin.

Recruitment and Enrollment

1. How would you modify the outreach and recruitment process to improve it or make it easier? (Probes: technical difficulties, scheduling challenge)
2. How comfortable were you with the recruitment, screening and enrollment process?
3. How can we improve the current screening process?
4. Would you prefer the recruitment and enrollment process to be online or in-person? Please explain why. (Probes: how found out about study, prior participation in research)
5. What motivated you to participate?
6. What considerations or concerns did you have before deciding to participate?
7. Would you consider participating in another research study in the future? Why or why not?

End of Interview

This is the end of the interview today. Before we wrap up, is there anything else you would like to share with me about your experience in the Smoking Cessation study?

Thank you for your time.
