# Supplementary material for: A Smoking Cessation Mobile App for Persons Living With HIV: Preliminary Efficacy and Feasibility Study
Source: JMIR Form Res. 2022 Aug 18;6(8):e28626. doi: 10.2196/28626 (PMC9437787; doi:10.2196/28626)
Supplement: Multimedia Appendix 4 [file formative_v6i8e28626_app4.pdf]

Smoking Cessation Pilot Baseline

Start of Block: Introduction

*

qs_id Smoking Cessation Pilot Survey - Baseline

User ID

____

Page Break

End of Block: Introduction

Start of Block: Demographics

Page Break

*

age What is your age (in years)?

____

*

dob What is your date of birth? (mm/dd/yyyy)

- Month (mm) (1) ____
- Day (dd) (2) ____
- Year (yyyy) (3) ____

X→

gender What is your current gender identity?

- O Male (1)
- O Female (2)
- O Transgender Male/Transman/FTM (3)
- O Transgender Female/Transwoman/MTF (4)
- O Genderqueer (5)
- O Other (please specify): (6) ____________________

X→

sex What sex were you assigned at birth?

- O Male (1)
- O Female (0)

X→

sexuality Which best describes your sexual orientation?

- O Homosexual/gay/lesbian (1)
- O Heterosexual/straight (2)
- O Bisexual (3)
- O Queer (4)
- O Asexual (5)
- O Other (please specify): (6) ____________________

X→

relationship What is your primary relationship status? Are you...

- O Single (1)
- O In a relationship with a man (2)
- O In a relationship with a woman (3)
- O Legally married to a man or in a registered civil union/domestic partnership with a man (4)
- O Legally married to a woman or in a registered civil union/domestic partnership with a woman (5)
- O Divorced/separated from a man (6)
- O Divorced/separated from a woman (7)
- O Widowed (male partner) (8)
- O Widowed (female partner) (9)
- O Other (please specify): (10) ____

Page Break

race Do you consider yourself: (Check all that apply)

- [ ] African American/Black (1)
- [ ] American Indian or Alaska Native (2)
- [ ] Asian (3)
- [ ] Native Hawaiian or Other Pacific Islander (4)
- [ ] White (5)
- [ ] Other (please specify): (6) ____________________

---

hispanic Do you consider yourself Hispanic/Latino?

- O Yes (1)
- O No (0)

---

spanish Are you fluent in Spanish?

- O Yes, I speak and read Spanish (1)
- O Yes, I speak Spanish (2)
- O Yes, I read Spanish (3)
- O No (4)

Page Break

education What is the highest degree or level of school you have completed?

- None (1)
- Elementary school (2)
- Some high school, no diploma (3)
- High school diploma or equivalent (e.g., GED) (4)
- Some college (5)
- Associate degree or technical degree (6)
- Bachelor/college degree (7)
- Professional or graduate degree (8)

work What is your current employment status? (Check all that apply)

- ☐ Working full-time (1)
- ☐ Working part-time (including seasonal, work-study, etc.) (2)
- ☐ Working off the books (not reported as taxable income) (3)
- ☐ Unemployed, looking for work (4)
- ☐ Unemployed, not looking for work (5)
- ☐ Retired (6)
- ☐ Student (7)
- ☐ Disabled (8)

income_annual My annual income (from all sources) is:

- O Less than $10,000 (1)
- O $10,000-$19,999 (2)
- O $20,000-$39,999 (3)
- O $40,000-$59,999 (4)
- O $60,000-$79,999 (5)
- O $80,000-$99,999 (6)
- O $100,000-$149,999 (7)
- O $150,000 or more (8)
- O Don't know (9)

money_notenough In the **last 12 months**, how many times was there NOT enough money in the household for rent, food, or utilities (e.g., gas or electric bill)?

- Never (0)
- 1 time (1)
- 2 times (2)
- 3 times (3)
- 4 times (4)
- 5 times (5)
- 6 times (6)
- 7 times (7)
- 8 times (8)
- 9 times (9)
- 10 times (10)
- 11 times (11)
- 12 times (12)
- More than 12 times (13)
- I don't know (14)

moving In the **last 12 months**, how many times did you move?

- O 0 (0)
- O 1 (1)
- O 2 (2)
- O 3 or more times (3)

---

children Do you have children?

- O Yes (1)
- O No (2)
- O Other (please describe): (3) ________________

Page Break

height About how tall are you without shoes?

O 4' 09" or less (1)

O 4' 10" (2)

O 4' 11" (3)

O 5' 00" (4)

O 5' 01" (5)

O 5' 02" (6)

O 5' 03" (7)

O 5' 04" (8)

O 5' 05" (9)

O 5' 06" (10)

O 5' 07" (11)

O 5' 08" (12)

O 5' 09" (13)

O 5' 10" (14)

O 5' 11" (15)

O 6' 00" (16)

O 6' 01" (17)

O 6' 02" (18)

O 6' 03" (19)

O 6' 04" (20)

O 6' 05" (21)

O 6' 06" (22)

O 6' 07" or more (23)

weight About how much do you weigh (in pounds) without shoes?

____

Page Break

healthinsur Do you currently have health insurance? (Check all that apply)

- ▢ Yes - Through my job (1)
- ▢ Yes - Through someone else's job (2)
- ▢ Yes - Through a health exchange (Affordable Care Act) (3)
- ▢ Yes - Paid for by me or another person (4)
- ▢ Yes - Medicaid/Medicare (5)
- ▢ Yes - ADAP (6)
- ▢ Yes - Veteran's Association (7)
- ▢ Other (please specify): (8) ____
- ▢ ⊗No (9)
- ▢ ⊗I don't know (10)

X→

med_forms How confident are you filling out medical forms by yourself?

- O Extremely (1)
- O Quite a bit (2)
- O Somewhat (3)
- O A little bit (4)
- O Not at all (5)

Page Break

diag3mo In the **last 3 months**, were you diagnosed with any of the following? (Check all that apply)

- ☐ Chancroid (1)
- ☐ Chlamydia (2)
- ☐ Gonorrhea (3)
- ☐ Hep A (4)
- ☐ Hep B (5)
- ☐ Hep C (6)
- ☐ Herpes - Genital (7)
- ☐ HPV - Genital/Anal Warts (8)
- ☐ Lymphogranuloma Venereum (9)
- ☐ Non-Gonococcal Urethritis (10)
- ☐ Syphilis (11)
- ☐ ⊗None of the above (0)

Page Break

disease Has a doctor or other medical provider **ever** told you that you had any of the following diseases or conditions? (Check all that apply)

- ☐ PCP (Pneumocystis Carinii Pneumonia) (1)
- ☐ Recurrent bacterial pneumonia (2)
- ☐ Cryptococcus (i.e., cryptococcal meningitis) (3)
- ☐ MAC (Disseminated Mycobacterium Avium Complex Disease) (4)
- ☐ Toxoplasmosis (infection of the brain) (5)
- ☐ Shingles (6)
- ☐ Recurrent herpes (HSV) or serious HSV infection (i.e., pneumonia) (7)
- ☐ CMV (cytomegalovirus) (8)
- ☐ Thrush (candidiasis) (9)
- ☐ Esophagitis (due to Candida, herpes, or CMV) (10)
- ☐ Retinitis (11)
- ☐ Tuberculosis (12)
- ☐ PML (Progressive multifocal leukoencephalopathy) (13)
- ☐ Lymphoma (14)
- ☐ KS (Kaposi's Sarcoma) (15)
- ☐ Anal cancer (16)
- ☐ Coccidioidomycosis (17)

- [ ] Histoplasmosis (18)
- [ ] Salmonella septicemia (recurrent) (19)
- [ ] Wasting syndrome from HIV (20)
- [ ] Encephalopathy (21)
- [ ] ⊗None of the above (0)

Page Break

diag_hana Have you **ever** been diagnosed with any of the following? (Check all that apply)

- [ ] Arthritis (1)
- [ ] Asthma (2)
- [ ] Cardiovascular disease (3)
- [ ] Chronic obstructive pulmonary disease (COPD) (4)
- [ ] Diabetes (5)
- [ ] Liver Disease (6)
- [ ] Osteoporosis (7)
- [ ] Renal failure (8)

diag Have you **ever** been diagnosed with any of the following? (Check all that apply)

- ☐ Alcohol abuse or dependence (1)
- ☐ Anxiety (2)
- ☐ Asperger's Syndrome or Autism Spectrum Disorder (3)
- ☐ Attention Deficit Disorder/ADHD (4)
- ☐ Bipolar or other mood disorder (5)
- ☐ Cancer (6)
- ☐ Chronic sinusitis (7)
- ☐ COPD (Chronic Obstructive Pulmonary Disease) or emphysema (8)
- ☐ Depression (9)
- ☐ Diabetes (10)
- ☐ Drug abuse or dependence (11)
- ☐ Eating disorder (anorexia, bulimia) (12)
- ☐ Epilepsy (13)
- ☐ Heart disease (14)
- ☐ High cholesterol (15)
- ☐ Hypertension (16)
- ☐ Insomnia (17)
- ☐ Low testosterone (18)

MRSA (Methicillin-Resistant Staphylococcus Aureus) (19)

Obsessive-Compulsive Disorder (20)

Parkinson's Disease (21)

Personality Disorder (22)

Post-Traumatic Stress Disorder (23)

Sexual dysfunction (24)

Schizophrenia (25)

Other (please specify): (26) ____

⊗None of the above (0)

Page Break

meds Are you **currently** taking any medications prescribed by your doctors? (Check all that apply)

- ▢ Antibiotics (e.g., Bactrim) (1)
- ▢ Anxiety medication (2)
- ▢ Bipolar medication (3)
- ▢ Blood pressure medication (4)
- ▢ Cancer medication or treatment (5)
- ▢ Cholesterol medication (6)
- ▢ Contraceptive pill (7)
- ▢ Depression medication (8)
- ▢ Diabetes medication (9)
- ▢ Erection medication (e.g., Viagra) (10)
- ▢ Heart medication (11)
- ▢ Herpes medication (12)
- ▢ Hormone replacement therapy (e.g., Premarin, Estrogel) (13)
- ▢ Schizophrenia medication (14)
- ▢ Seizure medication (15)
- ▢ Steroids (16)
- ▢ Testosterone (17)

- [ ] Other (please specify): (18) ________________

- [ ] ⊗None of the above (0)

Page Break

x→

care Where do you **usually** go for HIV-related health care? (Check all that apply)

- [ ] Community-based clinic (1)
- [ ] Urgent care clinic (2)
- [ ] Emergency room (3)
- [ ] Hospital (4)
- [ ] Mobile van (5)
- [ ] Private doctor's office (6)
- [ ] School/college clinic (7)
- [ ] ⊗I don't have regular source of health care (8)
- [ ] ⊗I don't seek health care (9)
- [ ] Other (please specify): (10) ________________

x→

provider Is there one doctor, nurse or other medical provider whom you consider to be in charge of your overall HIV health care now?

- O Yes (1)
- O No (0)

---

last_appt When was the last time you had a health care appointment with the medical provider you consider to be in charge of your overall HIV health care?

- O Last 3 months (1)
- O 3-6 months ago (2)
- O 6-9 months ago (3)
- O 9-12 months ago (4)
- O More than a year ago (5)
- O I don't know (6)

Page Break

vload_test_6mo In the **last 6 months**, did you have a viral load test?

- O Yes (1)
- O No (2)
- O I don't know (3)

---

vload_result What was the result of your most recent viral load test?

- My viral load was undetectable, OR (1)
- My viral load was detectable, OR >200 copies/ml (2)
- I don't know -- but I think I was detectable (3)
- I don't know -- but I think I was undetectable (4)

Page Break

cd4_test_6mo In the **last 6 months**, did you have a CD4 or T-cell test?

- Yes (1)
- No (2)
- I don't know (3)

cd4_result What was your **most recent** CD4 or T-cell count?

- Less than 200 (1)
- 201 - 349 (2)
- 350 - 499 (3)
- 500 or higher (4)
- I don't know (5)

Page Break

cd4_lowest When did your **lowest** CD4 or T-cell count occur? Please provide the month and year.

- Month (mm) (1) ____
- Year (yyyy) (2) ____

x→

cd4_lowestresult What was your **lowest** CD4 or T-cell count **ever**?

- Less than 200 (1)
- 201 - 349 (2)
- 350 - 499 (3)
- 500 or higher (4)
- I don't know (5)

Page Break

*

hivdx When were you diagnosed with HIV? Please provide the month and year.

- Month (mm) (1) ____
- Year (yyyy) (2) ____

x→

art Are you **currently** taking antiretroviral medications to treat your HIV infection?

- Yes (1)
- No (0)

Page Break

mh1 Over the **last 2 weeks**, how often were you bothered by feeling nervous, anxious, or on edge?

- Not at all (0)
- Several days (1)
- More than half the days (2)
- Nearly every day (3)

mh2 Over the **last 2 weeks**, how often were you bothered by not being able to stop or control worrying?

- Not at all (0)
- Several days (1)
- More than half the days (2)
- Nearly every day (3)

mh3 Over the **last 2 weeks**, how often were you bothered by little interest or pleasure in doing things?

- O Not at all (0)
- O Several days (1)
- O More than half the days (2)
- O Nearly every day (3)

X→

mh4 Over the **last 2 weeks**, how often were you bothered by feeling down, depressed or hopeless?

- O Not at all (0)
- O Several days (1)
- O More than half the days (2)
- O Nearly every day (3)

X→

mh5 Over the **last 2 weeks**, how often were you bothered by thoughts that you would be better off dead or of hurting yourself in some way?

- O Not at all (0)
- O Several days (1)
- O More than half the days (2)
- O Nearly every day (3)

Page Break

trap1 Please select the number 4 below. This helps prevent automated programs from abusing the study.

- O 6 (1)
- O 5 (2)
- O 4 (3)
- O 3 (4)
- O 2 (5)
- O 1 (6)

End of Block: Demographics

Start of Block: Medical History

X→

HTND Do you have high blood pressure or hypertension?

- O No (0)
- O Yes (1)

Display This Question:

If HTND = 1

*

HTNSTA At what age were you first told you had high blood pressure?

____

X→

MI Have you ever been told you had a heart attack?

- No (0)
- Yes, more than 6 months ago (1)
- Yes, within the last 6 months (2)
- Yes, unclear when (3)

CAD1 Have you ever had chest, jaw, or arm pains which worsen with exercise (angina)?

- No (0)
- Yes (1)

CAD2 Are you currently taking medicines for a heart condition?

- No (0)
- Yes (1)

CAD3 Have you ever had bypass surgery or balloon angioplasty for your heart?

- No (0)
- Yes (1)

Page Break

CHF1 Have you ever been told you had heart failure?

- O No (0)
- O Yes (1)

CHF2 Do you get shortness of breath with minimal exertion or while sleeping?

- O No (0)
- O Yes (1)

AF Have you ever been told you had atrial fibrillation?

- O No (0)
- O Yes (1)

Page Break

DM Do you have diabetes or high sugar?

- O No (0)
- O Yes, diet advised (1)
- O Yes, oral medicines (2)
- O Yes, insulin (3)

Display This Question:

If DM != 0

*

DMSTA At what age were you first told you had diabetes or high sugar?

____

X→

HCOL Have you ever been told you have high cholesterol?

- No (0)
- Yes, not treated (1)
- Yes, diet advised (2)
- Yes, taking meds (3)

Display This Question:

If HCOL != 0

*

CHOLSTA At what age were you first told you had high cholesterol?

____

End of Block: Medical History

Start of Block: Tobacco Use History (part 1)

cigarette_use Which best describes your **current** cigarette use?

- Regular smoker (1)
- Occasional smoker (2)
- I don't smoke (3)

---

cigs_filter Do you usually smoke filtered or unfiltered cigarettes?

- Filtered (1)
- Unfiltered (2)

---

cigs_brand What is your usual brand of cigarettes?

____

End of Block: Tobacco Use History (part 1)

Start of Block: Tobacco Use History (part 2)

cig_type Do you usually smoke menthol or non-menthol cigarettes?

- Menthol (1)
- Non-Menthol (2)
- Other (3) ____
- No usual type (4)

Page Break

Display This Question:

If cig_type = 1

Or cig_type = 2

cig_daysperweek How many days per week do you smoke?

____

Display This Question:

If cig_type = 1

Or cig_type = 2

cig_cigsperday How many cigarettes per day do you smoke?

____

Display This Question:

If cig_type = 1

Or cig_type = 2

cig_totalyears For how many years have you been smoking?

____

Page Break

cig_othertype In the past 30 days, have you used any of the following:

	Yes (1)	No (2)
Electronic cigarettes (1)	O	O
Other tobacco products/little cigars/cigarillos (2)	O	O

Page Break

*

cig_agefirstcig How old were you when you smoked your first cigarette?

____

*

cig_ageregularuse At what age did you first start to smoke cigarettes regularly or most days?

____

Page Break

cigs_everquit Have you EVER tried to QUIT smoking COMPLETELY, even if you made it less than one day?

O Yes (1)

O No (2)

*

cigs_everquittimes How many times have you EVER tried to QUIT smoking completely?

____

Page Break

cigs_12moquit During the past 12 months, have you tried to QUIT smoking COMPLETELY, even if you made it less than one day?

- O Yes (1)
- O No (2)

Page Break

Display This Question:

If cigs_12moquit = 1

cigs_12moquittimes During the past 12 months when you were trying to quit smoking, how many times were you able to stop smoking for one day or longer?

____

Display This Question:

If cigs_12moquit = 1

cigs_12moquittime During the past 12 months when you were trying to quit smoking, what was the LONGEST amount of time you stopped smoking?

- O Days: (1) ____
- O Weeks: (2) ____
- O Months: (3) ____

Page Break

cigs_90dayquit During the past 90 days (or 3 months), have you tried to QUIT smoking COMPLETELY?

- O Yes (1)
- O No (2)

---

cigs_evercutdown Have you **ever** tried to cut down on the number of cigarettes you smoke per day?

- O Yes (1)
- O No (2)

Page Break

cigs_otheryr Have you used any other **smokable** tobacco products in the past year? Select all that apply.

- [ ] Cigars (1)
- [ ] Cigarillos (e.g. Black and Mild) (2)
- [ ] Little Cigars (e.g. Winchester) (4)
- [ ] Pipe filled with tobacco (5)
- [ ] Hookah (6)
- [ ] Bidis (7)
- [ ] Clove Cigarettes (8)
- [ ] Marijuana/Tobacco Combinations (e.g. spliff, blunt) (9)
- [ ] Other (please specify): (10) ____
- [ ] ⊗I did not use any other smokable tobacco products in the past year (11)

cigs_smklessyr Have you used any other **smokeless** tobacco products in the past year? Select **all** that apply.

- ☐ Chewing Tobacco (e.g. Redman) (1)
- ☐ Snuff (e.g. Skoal) (2)
- ☐ Snus (e.g. Camel Snus) (3)
- ☐ Dissolvables (e.g. Camel Orbs) (4)
- ☐ Other (please specify): (5) ____
- ☐ ⊗I did not use any other tobacco products in the past year (6)

Page Break

Display This Question:

If cigs_otheryr != 11

cigs_otheryrday In the past year, on how many days did you use other **smokable** tobacco products?

____

Display This Question:

If cigs_otheryr != 11

cigs_otheryrdrow In the past year, how many of those days were in a row?

____

Page Break

Display This Question:

If cigs_smklessyr != 6

*

cigs_smklessyrday In the past year, on how many days did you use other **smokeless** tobacco products?

____

Display This Question:

If cigs_smklessyr != 6

*

cigs_smklessyrdrow In the past year, how many of those days were in a row?

____

Page Break

Display This Question:

If cigs_otheryr != 11

X→

cigs_other_30days Have you used any other **smokable** tobacco products in the past 30 days? Select all that apply.

- [ ] Cigars (1)
- [ ] Cigarillos (e.g. Black and Mild) (2)
- [ ] Little Cigars (e.g. Winchester) (4)
- [ ] Pipe filled with tobacco (5)
- [ ] Hookah (6)
- [ ] Bidis (7)
- [ ] Clove Cigarettes (8)
- [ ] Marijuana/Tobacco Combinations (e.g. spliff, blunt) (9)
- [ ] Other (please specify): (10) ____
- [ ] ⊗I did not use any other smokable tobacco products in the past 30 days (11)

Display This Question:

If cigs_smklessyr != 6

X→

cigs_smkless_30days Have you used any other **smokeless** tobacco products in the past 30 days? Select **all** that apply.

- ☐ Chewing Tobacco (e.g. Redman) (1)
- ☐ Snuff (e.g. Skoal) (2)
- ☐ Snus (e.g. Camel Snus) (3)
- ☐ Dissolvables (e.g. Camel Orbs) (4)
- ☐ Other (please specify): (5) ____
- ☐ ⊗I did not use any other tobacco products in the past 30 days (6)

Page Break

Display This Question:

If cigs_otheryr != 11

And cigs_other_30days != 11

cigs_othermnthday In the past 30 days, on how many days did you use other **smokable** tobacco products?

____

Display This Question:

If cigs_otheryr != 11

And cigs_other_30days != 11

cigs_othermnthdrow In the past 30 days, how many of those days were in a row?

____

Page Break

Display This Question:

If cigs_smklessyr != 6

And cigs_smkless_30days != 6

*

cigs_smklessmnthday In the past 30 days, on how many days did you use other **smokeless** tobacco products?

____

Display This Question:

If cigs_smklessyr != 6

And cigs_smkless_30days != 6

*

cigs_smklessmnthdrow In the past 30 days, how many of those days were in a row?

____

Page Break

Display This Question:

If cigs_otheryr != 11

And cigs_other_30days != 11

cigs_otherlasttime When was the last time you used other **smokable** tobacco products? This includes cigars, cigarillos, blunts, spliffs, etc.

O Month (MM) (1) ____

O Day (DD) (2) ____

O Year (YYYY) (3) ____

Display This Question:

If cigs_smklessyr != 6

And cigs_smkless_30days != 6

cigs_smklesslasttime When was the last time you used other **smokeless** tobacco products? This includes chew, snus, snuff, etc.

- Month (MM) (1) ____
- Day (DD) (2) ____
- Year (YYYY) (3) ____

X→

mj_smoke_past30 In the past 30 days, have you smoked marijuana, K2/spice, or any other synthetic cannabinoids? (e.g. smoking a joint, pipe, bong, etc.)

- Yes (1)
- No (0)

Page Break

cigs_quitmethod Please answer the following.

	Have you EVER used the following products for the purpose of stopping smoking? (please enter YES or NO) (1)	If YES, when was the last time you used it? (MM/DD/YYYY) (2)
Nicotine Patch (1)		
Nicotine Gum (2)		
Nicotine lozenge, inhaler, or nasal spray (3)		
E-cigarette (4)		
Bupropion (Wellbutrin, Zyban) (5)		
Varenicline (Chantix) (6)		
Behavioral (talk) therapy (7)		
Other products, medicine, or treatments (List, if applicable) (8)		

cigs_quitline Have you ever used a quitline or telephone counseling service to help you quit smoking?

O Yes (1)

O No (2)

Page Break

Display This Question:

If cigs_quitline = 1

cigs_quitlinetime When was the last time you used a quitline or telephone counseling service?

O Month (mm) (1) ____

O Day (dd) (2) ____

O Year (yyyy) (3) ____

End of Block: Tobacco Use History (part 2)

Start of Block: Alcohol History & CAGE Questions

ETEVER In your lifetime did you ever drink wine, beer, or liquor?

O No (0)

O Yes (1)

Skip To: End of Block If ETEVER = 0

Page Break

ETSTA How old were you when you started?

____

ETSTO How old were you when you stopped?
If you still drink, then enter your current age.

____

Page Break

ETAVG On a day in which you drank alcohol, how many drinks on average did you have?

____

ETTYPE What do *or did* you usally drink?

- O Beer (1)
- O Wine (2)
- O Liquor (3)
- O More than 1 (4)

ETLBEER When you drank what was the **average** amount of **beer** you drank in terms of bottles, cans, or glasses? If none, enter 0.

____

ETLWINE When you drank what was the **average** amount of **wine** you drank in terms of 4 ounce glasses? If none, enter 0.

____

ETLLIQ When you drank what was the **average** amount of **liquor** you drank (whiskey, gin, vodka, etc.) in terms of drinks where a drink has 1 shot of liquor? If none, enter 0.

____

Page Break

ETOH In the last year did you drink **any** wine, beer, or liquor?

- No (0)
- Yes (1)

Skip To: ETPART If ETOH = 0

ETBEER During the past year, what was the **average** amount of **beer** you drank in terms of bottles, cans, or glasses? If none, enter 0.

____

ETWINE During the past year, what was the **average** amount of **wine** you drank in terms of 4 ounce glasses? If none, enter 0.

____

ETLIQ During the past year, what was the **average** amount of **liquor** you drank (whiskey, gin, vodka, etc.) in terms of drinks where a drink has 1 shot of liquor? If none, enter 0.

____

Page Break

ETMAX In the last year what was the **maximum** number of drinks you had per day?

____

Page Break

ET24H In the last 24 hours, how many drinks did you have?

- O None (0)
- O 1 drink (1)
- O 2-3 drinks (2)
- O 4-5 drinks (3)
- O 6 or more (4)

ETPART Does your significant other ever drink?

O No (0)

O Yes (1)

Page Break

X→

CUT Have you ever felt you should **CUT** down on your drinking?

O No (0)

O Yes (1)

X→

ANNOYED Have people **ANNOYED** you by criticizing your drinking?

O No (0)

O Yes (1)

X→

GUILTY Have you ever felt bad or **GUILTY** about your drinking?

O No (0)

O Yes (1)

X→

EYE Have you ever had a drink first thing in the morning to steady your nerves or get rid of a hangover (**EYE**-opener)?

O No (0)

O Yes (1)

Page Break

ETHPAST1 Over the last 20 years, was there ever a period when you drank on average **more than** 2 beers, glasses of wine, or drinks **per day**?

O No (0)

O Yes (1)

Page Break

Display This Question:

If ETHPAST1 = 1

ETHPAST2 For how many years did this period last?

____

ALCON Have you ever been hospitalized for an alcohol-related condition?
(Such as detox, cirrhosis, DTs, esophageal bleeding, or alcoholic hepatitis)

O No (0)

O Yes (1)

End of Block: Alcohol History & CAGE Questions

Start of Block: Short Michigan Alcoholism Screening Test (SMAST)

x→

current_drink Do you currently drink (regularly or casually)?

O Yes (1)

O No (2)

Skip To: End of Block If current_drink = 2

Page Break

smast1 Do you feel you are a normal drinker?
By normal we mean you drink less than or as much as most other people.

O Yes (1)

O No (2)

smast2 Does your wife, husband, a parent, or other near relative every worry or complain about your drinking?

O Yes (1)

O No (2)

smast3 Do you ever feel guilty about your drinking?

O Yes (1)

O No (2)

smast4 Do friends or relatives think you are a normal drinker?

- O Yes (1)
- O No (2)

smast5 Are you able to stop drinking when you want to?

- O Yes (1)
- O No (2)

Page Break

smast6 Have you ever attended a meeting of Alcoholics Anonymous?

- O Yes (1)
- O No (2)

smast7 Has drinking ever created problems between you and your wife, husband, a parent, or other near relative?

- O Yes (1)
- O No (2)

smast8 Have you ever gotten into trouble at work or school because of drinking?

- O Yes (1)
- O No (2)

smast9 Have you ever neglected your obligations, your family, or your work for two or more days in a row because you were drinking?

O Yes (1)

O No (2)

X→

smast10 Have you ever gone to anyone for help about your drinking?

O Yes (1)

O No (2)

Page Break

Display This Question:

If smast10 = 1

smast10a Was this other than Alcoholics Anonymous or a hospital?

O Yes (1)

O No (2)

X→

smast11 Have you ever been in a hospital because of drinking?

O Yes (1)

O No (2)

Skip To: smast12 If smast11 = 2

Page Break

smast11a Was this for detox?

- O Yes (1)
- O No (2)

smast11b Was this for alcoholism treatment?

- O Yes (1)
- O No (2)

smast11c Was this for alcohol-related injuries or medical problems, e.g., cirrhosis or physical injury incurred while under the influence of alcohol (car accident, fight, etc.)?

- O Yes (1)
- O No (2)

Page Break

smast12 Have you ever been arrested for drunken driving, driving while intoxicated, or driving under the influence of alcoholic beverages?

- O Yes (1)
- O No (2)

smast13 Have you ever been arrested, even for a few hours, because of other drunken behavior?

- O Yes (1)
- O No (2)

End of Block: Short Michigan Alcoholism Screening Test (SMAST)

Start of Block: NIDA ASSIST

X→

nida1
This question refers to illicit or illegal drug use as well as prescription drugs taken for reasons or in doses other than prescribed.

In your <u>lifetime</u>, which of the following substances have you ever used?

	Yes (2)	No (0)
**Cannabis** (marijuana, pot, etc.) (nida1_1)	O	O
**Cocaine** (coke, crack, etc.) (nida1_2)	O	O
**Prescription stimulants** (Ritalin, Adderall, diet pills, etc.) (nida1_3)	O	O
**Methamphetamine** (speed, crystal meth, etc.) (nida1_4)	O	O
**Inhalants** (nitrous oxide, glue, gas, etc.) (nida1_5)	O	O
**Sedatives or sleeping pills** (Valium, Ativan, Xanax, GHB, etc.) (nida1_6)	O	O
**Hallucinogens** (ecstasy, acid, mushrooms, etc.) (nida1_7)	O	O
**Street opioids** (heroin, opium, etc.) (nida1_8)	O	O
**Prescription opioids** (Percocet, Vicodin, methadone, etc.) (nida1_9)	O	O
**Other** (please specify): (nida1_10)	O	O

Page Break

nida2 This question refers to illicit or illegal drug use as well as prescription drugs taken for reasons or in doses <u>other than prescribed</u>.

In the **past three months**, how often have you used.... ?

	Never (0)	Once or twice (2)	Monthly (3)	Weekly (4)	Daily or almost daily (6)
**Cannabis** (marijuana, pot, etc.) (1)	O	O	O	O	O
**Cocaine** (coke, crack, etc.) (2)	O	O	O	O	O
**Prescription stimulants** (Ritalin, Adderall, diet pills, etc.) (3)	O	O	O	O	O
**Methamphetamine** (speed, crystal meth, etc.) (4)	O	O	O	O	O
**Inhalants** (nitrous oxide, glue, gas, etc.) (5)	O	O	O	O	O
**Sedatives or sleeping pills** (Valium, Ativan, Xanax, GHB, etc.) (6)	O	O	O	O	O
**Hallucinogens** (ecstasy, acid, mushrooms, etc.) (7)	O	O	O	O	O
**Street opioids** (heroin, opium, etc.) (8)	O	O	O	O	O
**Prescription opioids** (Percocet, Vicodin, methadone, etc.) (9)	O	O	O	O	O
**Other** (please specify): (10)	O	O	O	O	O

nida3

In the past 3 months, how often have you had a strong desire or urge to use each of the following?

	Never (0)	Once or Twice (3)	Monthly (4)	Weekly (5)	Daily or Almost Daily (6)
**Cannabis** (marijuana, pot, etc.) (1)	O	O	O	O	O
**Cocaine** (coke, crack, etc.) (2)	O	O	O	O	O
**Prescription stimulants** (Ritalin, Adderall, diet pills, etc.) (3)	O	O	O	O	O
**Methamphetamine** (speed, crystal meth, etc.) (4)	O	O	O	O	O
**Inhalants** (nitrous oxide, glue, gas, etc.) (5)	O	O	O	O	O
**Sedatives or sleeping pills** (Valium, Ativan, Xanax, GHB, etc.) (6)	O	O	O	O	O
**Hallucinogens** (ecstasy, acid, mushrooms, etc.) (7)	O	O	O	O	O
**Street opioids** (heroin, opium, etc.) (8)	O	O	O	O	O
**Prescription opioids** (Percocet, Vicodin, methadone, etc.) (9)	O	O	O	O	O
**Other** (please specify): (10)	O	O	O	O	O

nida4

During the past 3 months, how often has your use of each of the following drugs led to health, social, legal or financial problems?

	Never (0)	Once or Twice (4)	Monthly (5)	Weekly (6)	Daily or Almost Daily (7)
**Cannabis** (marijuana, pot, etc.) (1)	O	O	O	O	O
**Cocaine** (coke, crack, etc.) (2)	O	O	O	O	O
**Prescription stimulants** (Ritalin, Adderall, diet pills, etc.) (3)	O	O	O	O	O
**Methamphetamine** (speed, crystal meth, etc.) (4)	O	O	O	O	O
**Inhalants** (nitrous oxide, glue, gas, etc.) (5)	O	O	O	O	O
**Sedatives or sleeping pills** (Valium, Ativan, Xanax, GHB, etc.) (6)	O	O	O	O	O
**Hallucinogens** (ecstasy, acid, mushrooms, etc.) (7)	O	O	O	O	O
**Street opioids** (heroin, opium, etc.) (8)	O	O	O	O	O
**Prescription opioids** (Percocet, Vicodin, methadone, etc.) (9)	O	O	O	O	O
**Other** (please specify): (10)	O	O	O	O	O

nida5

During the past 3 months, how often have you failed to do what was normally expected of you because of your use of each of the following drugs?

	Never (0)	Once or Twice (5)	Monthly (6)	Weekly (7)	Daily or Almost Daily (8)
**Cannabis** (marijuana, pot, etc.) (1)	O	O	O	O	O
**Cocaine** (coke, crack, etc.) (2)	O	O	O	O	O
**Prescription stimulants** (Ritalin, Adderall, diet pills, etc.) (3)	O	O	O	O	O
**Methamphetamine** (speed, crystal meth, etc.) (4)	O	O	O	O	O
**Inhalants** (nitrous oxide, glue, gas, etc.) (5)	O	O	O	O	O
**Sedatives or sleeping pills** (Valium, Ativan, Xanax, GHB, etc.) (6)	O	O	O	O	O
**Hallucinogens** (ecstasy, acid, mushrooms, etc.) (7)	O	O	O	O	O
**Street opioids** (heroin, opium, etc.) (8)	O	O	O	O	O
**Prescription opioids** (Percocet, Vicodin, methadone, etc.) (9)	O	O	O	O	O
**Other** (please specify): (10)	O	O	O	O	O

nida6
Has a friend or relative or anyone else ever expressed concern about your use of each of the following?

	No, never (0)	Yes, but not in the past 3 months (3)	Yes, in the past 3 months (6)
**Cannabis** (marijuana, pot, etc.) (1)	o	o	o
**Cocaine** (coke, crack, etc.) (2)	o	o	o
**Prescription stimulants** (Ritalin, Adderall, diet pills, etc.) (3)	o	o	o
**Methamphetamine** (speed, crystal meth, etc.) (4)	o	o	o
**Inhalants** (nitrous oxide, glue, gas, etc.) (5)	o	o	o
**Sedatives or sleeping pills** (Valium, Ativan, Xanax, GHB, etc.) (6)	o	o	o
**Hallucinogens** (ecstasy, acid, mushrooms, etc.) (7)	o	o	o
**Street opioids** (heroin, opium, etc.) (8)	o	o	o
**Prescription opioids** (Percocet, Vicodin, methadone, etc.) (9)	o	o	o
**Other** (please specify): (10)	o	o	o

Page Break

nida7
Have you ever tried and failed to control, cut down or stop using each of the following?

	No, never (0)	Yes, but not in the past 3 months (3)	Yes, in the past 3 months (6)
**Cannabis** (marijuana, pot, etc.) (1)	o	o	o
**Cocaine** (coke, crack, etc.) (2)	o	o	o
**Prescription stimulants** (Ritalin, Adderall, diet pills, etc.) (3)	o	o	o
**Methamphetamine** (speed, crystal meth, etc.) (4)	o	o	o
**Inhalants** (nitrous oxide, glue, gas, etc.) (5)	o	o	o
**Sedatives or sleeping pills** (Valium, Ativan, Xanax, GHB, etc.) (6)	o	o	o
**Hallucinogens** (ecstasy, acid, mushrooms, etc.) (7)	o	o	o
**Street opioids** (heroin, opium, etc.) (8)	o	o	o
**Prescription opioids** (Percocet, Vicodin, methadone, etc.) (9)	o	o	o
**Other** (please specify): (10)	o	o	o

nida8 Have you **ever** used any drug by injection? (Nonmedical use only)

- O Yes, in the past 3 months (1)
- O Yes, but not in the past 3 months (2)
- O No (3)

End of Block: NIDA ASSIST

Start of Block: DAST

dastinstruct The following questions concern information about your involvement with drugs. Drug abuse refers to (1) the use of prescribed or “over-the-counter” drugs in excess of the directions, and (2) any non-medical use of drugs.

Consider the **past year** (*12 months*) and carefully read each statement. Then decide whether your answer is YES or NO and check the appropriate space. Please be sure to answer every question.

---

dast1 Have you used drugs other than those required for medical reasons?

- O Yes (1)
- O No (0)

---

dast2 Have you abused prescription drugs?

- O Yes (1)
- O No (0)

Skip To: End of Block If dast2 = 0

Page Break

dast3 Do you abuse more than one drug at a time?

- Yes (1)
- No (2)

---

dast4 Can you get through the week without using drugs (other than those required for medical reasons)?

- Yes (0)
- No (1)

---

dast5 Are you always able to stop using drugs when you want to?

- Yes (0)
- No (1)

---

dast6 Do you abuse drugs on a continuous basis?

- Yes (1)
- No (2)

---

dast7 Do you try to limit your drug use to certain situations?

- Yes (0)
- No (1)

Page Break

dast8 Have you had “blackouts” or “flashbacks” as a result of drug use?

- Yes (1)
- No (2)

dast9 Do you ever feel bad about your drug abuse?

- Yes (1)
- No (2)

dast10 Does your spouse (or parents) ever complain about your involvement with drugs?

- Yes (1)
- No (2)

dast11 Do your friends or relatives know or suspect you abuse drugs?

- Yes (1)
- No (2)

dast12 Has drug abuse ever created problems between you and your spouse?

- O Yes (1)
- O No (2)

---

dast13 Has any family member ever sought help for problems related to your drug use?

- O Yes (1)
- O No (2)

---

dast14 Have you ever lost friends because of your use of drugs?

- O Yes (1)
- O No (2)

Page Break

dast15 Have you ever neglected your family or missed work because of your use of drugs?

- O Yes (1)
- O No (2)

---

dast16 Have you ever been in trouble at work because of drug abuse?

- O Yes (1)
- O No (2)

---

dast17 Have you ever lost a job because of drug abuse?

- O Yes (1)
- O No (2)

dast18 Have you gotten into fights when under the influence of drugs?

- O Yes (1)
- O No (2)

dast19 Have you ever been arrested because of unusual behavior while under the influence of drugs?

- O Yes (1)
- O No (2)

dast20 Have you ever been arrested for driving while under the influence of drugs?

- O Yes (1)
- O No (2)

dast21 Have you engaged in illegal activities in order to obtain drugs?

- O Yes (1)
- O No (2)

dast22 Have you ever been arrested for possession of illegal drugs?

O Yes (1)

O No (2)

Page Break

dast23 Have you ever experienced withdrawal symptoms as a result of heavy drug intake?

O Yes (1)

O No (2)

dast24 Have you had medical problems as a result of your drug use (e.g., memory loss, hepatitis, convulsions, bleeding, etc.)?

O Yes (1)

O No (2)

dast25 Have you ever gone to anyone for help for a drug problem?

O Yes (1)

O No (2)

dast26 Have you ever been in a hospital for medical problems related to your drug use?

O Yes (1)

O No (2)

dast27 Have you ever been involved in a treatment program specifically related to drug use?

- O Yes (1)
- O No (2)

---

dast28 Have you been treated as an outpatient for problems related to drug abuse?

- O Yes (1)
- O No (2)

End of Block: DAST

Start of Block: Non-CTP Medical and Other Services

nms1 Are you **currently** receiving substance abuse treatment services?

- O Yes (1)
- O No (2)
- O Prefer not to answer (3)

X→

nms2
In the **past 30 days**, have you participated in an outpatient treatment program for drug or alcohol problems?

- O Yes (1)
- O No (2)
- O Prefer not to answer (3)

Skip To: nms3 If nms2 != 1

Page Break

nonctp_instructions1 These questions refer to your participation in outpatient treatment program(s) for drug or alcohol problems within the **last 30 days**.

nms2a How many days have you participated?

____

nms2b How many hours do you attend the program in a typical week?

____

Page Break

nms3
In the **past 30 days**, have you been admitted into a residential program for detox or for other services?

- O Yes (1)
- O No (2)
- O Prefer not to answer (3)

Skip To: nms4 If nms3 != 1

Page Break

nonctp_instructions2 These questions refer to your admission(s) the residential programs for detox or for other services within the **last 30 days**.

---

*

nms3a
How many admissions?

____

---

*

nms3b
How many nights altogether for all stays?

____

Page Break

X→

nms4
In the **past 30 days**, have you been admitted into a hospital for detox?

O Yes (1)

O No (2)

O Prefer not to answer (3)

Skip To: nms5 If nms4 != 1

Page Break

nonctp_instructions3 These questions refer to your admission(s) into a hospital for detox within the **last 30 days**.

---

*

nms4a
How many admissions?

____

---

*

nms4b
How many nights altogether for all stays?

____

Page Break

nms5
In the **past 30 days**, have you been admitted to the hospital for any other reason?

- O Yes (1)
- O No (2)
- O Prefer not to answer (3)

Skip To: nms6 If nms5 != 1

Page Break

nonctp_instructions4 These questions refer to your admission(s) into a hospital for any reason other than detox within the **last 30 days**.

---

nms5a
How many times were you admitted?

____

nms5b
How many nights altogether for all stays?

____

nms5c
Were any admissions for <u>psychiatric or emotional reasons</u>?

- O Yes (1)
- O No (2)
- O Prefer not to answer (3)

Page Break

nms6
In the **past 30 days**, have you visited an emergency room and not been admitted to the hospital?

- O Yes (1)
- O No (2)
- O Prefer not to answer (3)

Skip To: nms7 If nms6 != 1

Page Break

nonctp_instructions5 This question refers to your visit(s) to an emergency room without admission to the hospital within the **last 30 days**.

*

nms6a How many times did you visit the emergency room without being admitted to the hospital?

____

Page Break

nms7
Outside of the services or programs mentioned above, have you seen a therapist, that is a psychiatrist, psychologist, counselor, or social worker for psychological or emotional problems in the **last 30 days**?

- O Yes (1)
- O No (2)
- O Prefer not to answer (3)

Skip To: nms8 If nms7 != 1

Page Break

nonctp_instructions6 This question refers to your visit(s) with a therapist for psychological or emotional problems within the **last 30 days**.

*

nms7a
How many times did you see a psychiatrist, psychologist, counselor or social worker?

____

Page Break

nms8
Outside of the services or programs mentioned above, have you seen a therapist, that is a psychiatrist, psychologist, counselor, or social worker for alcohol or drug problems in the **last 30 days**?

- O Yes (1)
- O No (2)
- O Prefer not to answer (3)

Skip To: nms9 If nms8 != 1

Page Break

nonctp_instructions7 This question refers to your visit(s) with a therapist for alcohol or drug problems within the **last 30 days**.

nms8a
How many times did you see a psychiatrist, psychologist, counselor or social worker?

____

Page Break

nms9
In the **last 30 days**, have you visited a medical office, not including your therapist? (Please include all visits to a physician, nurse, nurse practitioner, or physician's assistant.)

- O Yes (1)
- O No (2)
- O Prefer not to answer (3)

Skip To: nms10 If nms9 != 1

Page Break

nonctp_instructions8 These questions refer to your visit(s) to a medical office within the **last 30 days**.

nms9a
How many visits to a medical office have you had?

____

nms9b
How many of these visits did you see a doctor?

____

Page Break

nms10
In the **last 30 days**, have you attended Alcoholics Anonymous (AA), Narcotics Anonymous (NA), or Cocaine Anonymous (CA) meetings?

O Yes (1)

O No (2)

O Prefer not to answer (3)

Skip To: nms11 If nms10 != 1

Page Break

nonctp_instructions9 This question refers to your attendance at AA, NA, or CA within the **last 30 days**.

*

nms10a
For how many days?

____

Page Break

nms11
Are you **currently** prescribed any medication for the treatment of substance abuse?

O Yes (1)

O No (2)

O Prefer not to answer (3)

Skip To: nms12 If nms11 != 1

Page Break

nonctp_instruction10 These questions refer to your **current** prescription(s) for medication for the treatment of substance abuse.

nms11a Which of the following medications have you been prescribed?

- ☐ Suboxone (1)
- ☐ Subutex/buprenorphine (2)
- ☐ Methadone (3)
- ☐ Naltrexone (4)
- ☐ Depot Naltrexone (5)
- ☐ Acamprosate (6)
- ☐ Antabuse/disulfiram (7)
- ☐ Other (please specify): (8) ____

nms11b In the **last 30 days**, how many total days did you take this/these medication(s)?

____

Page Break

nms12
In the **past 3 months**, have you spent time uninsured?

- O Yes (1)
- O No (2)
- O Prefer not to answer (3)

nms13 In the **last 30 days**, have you had a job? (This includes any job for which you have been paid, including under-the-table work.)

- O Yes (1)
- O No (2)
- O Prefer not to answer (3)

Skip To: End of Block If nms13 != 1

Page Break

nonctp_instruction11 These questions refer to the job(s) for which you have been paid, including under-the-table work, in the **past 30 days**.

nms13a
How many days have you been paid for working?

____

nms13b
Including overtime, how many hours per week do you work on this job?

____

nms13c
Including tips and bonuses, what is your hourly rate on this job, before taxes?

O $ (1) ____

End of Block: Non-CTP Medical and Other Services

Start of Block: PROMIS-29

X→

promis_pf **Physical Function**

	Without any difficulty (5)	With a little difficulty (4)	With some difficulty (3)	With much difficulty (2)	Unable to do (1)
Are you able to do chores such as vacuuming or yard work? (1)	O	O	O	O	O
Are you able to go up and down stairs at a normal pace? (2)	O	O	O	O	O
Are you able to go for a walk of at least 15 minutes? (3)	O	O	O	O	O
Are you able to run errands and shop? (4)	O	O	O	O	O

Page Break

promis_anx **Anxiety** **In the past 7 days…**

	Never (1)	Rarely (2)	Sometimes (3)	Often (4)	Always (5)
I felt fearful. (1)	O	O	O	O	O
I found it hard to focus on anything other than my anxiety. (2)	O	O	O	O	O
My worries overwhelmed me. (3)	O	O	O	O	O
I felt uneasy. (4)	O	O	O	O	O

promis_dep Depression In the past 7 days…

	Never (1)	Rarely (2)	Sometimes (3)	Often (4)	Always (5)
I felt worthless. (1)	O	O	O	O	O
I felt helpless. (2)	O	O	O	O	O
I felt depressed. (3)	O	O	O	O	O
I felt hopeless. (4)	O	O	O	O	O

Page Break

promis_fat
Fatigue

	Not at all (1)	A little bit (2)	Somewhat (3)	Quite a bit (4)	Very much (5)
**During the past 7 days…** I feel fatigued. (promis_fat_1)	O	O	O	O	O
I have trouble starting things because I am tired. (promis_fat_2)	O	O	O	O	O
**In the past 7 days…** How run-down did you feel on average? (promis_fat_3)	O	O	O	O	O
How fatigued were you on average? (promis_fat_4)	O	O	O	O	O

X→

promis_sd1 Sleep Disturbance In the past 7 days…

	Very poor (5)	Poor (4)	Fair (3)	Good (2)	Very good (1)
My sleep quality was... (promis_sd_1)	O	O	O	O	O

X→

promis_sd2 In the past 7 days…

	Very much (1)	Quite a bit (2)	Somewhat (3)	A little bit (4)	Not at all (5)
My sleep was refreshing. (promis_sd_2)	o	o	o	o	o

promis_sd3-4 In the past 7 days…

	Not at all (1)	A little bit (2)	Somewhat (3)	Quite a bit (4)	Very much (5)
I had a problem with my sleep. (promis_sd_3)	o	o	o	o	o
I had difficulty falling asleep. (promis_sd_4)	o	o	o	o	o

Page Break

promis_ssr

Satisfaction with Social Role

In the past 7 days…

	Not at all (1)	A little bit (2)	Somewhat (3)	Quite a bit (4)	Very much (5)
I am satisfied with how much work I can do (include work at home). (1)	o	o	o	o	o
I am satisfied with my ability to work (include work at home). (2)	o	o	o	o	o
I am satisfied with my ability to do regular personal and household responsibilities. (3)	o	o	o	o	o
I am satisfied with my ability to perform my daily routines. (4)	o	o	o	o	o

Page Break

promis_pi Pain Interference In the past 7 days…

	Not at all (1)	A little bit (2)	Somewhat (3)	Quite a bit (4)	Very much (5)
How much did pain interfere with your day to day activities? (1)	o	o	o	o	o
How much did pain interfere with work around the home? (2)	o	o	o	o	o
How much did pain interfere with your ability to participate in social activities? (3)	o	o	o	o	o
How much did pain interfere with your household chores? (4)	o	o	o	o	o

promis_painint Pain Intensity In the past 7 days…
How would you rate your pain on average?

O **No pain**0 (0)

O 1 (1)

O 2 (2)

O 3 (3)

O 4 (4)

O 5 (5)

O 6 (6)

O 7 (7)

O 8 (8)

O 9 (9)

O **Worstimaginable pain**10 (10)

End of Block: PROMIS-29

Start of Block: Symptom Distress Module

X→

sdm During the **past four weeks**, did you have.............?

	**No**, I did NOT HAVE THIS SYMPTOM (0)	Yes, I have this symptom. **It did NOT bother me** (1)	Yes, I have this symptom. **It bothered me a little** (2)	Yes, I have this symptom. **It bothered me** (3)	Yes, I have this symptom. **It bothered me a lot** (4)
Fatigue or loss of energy? (sdm_1)	O	O	O	O	O
Fevers, chills or sweats? (sdm_2)	O	O	O	O	O
Feeling dizzy or lightheaded? (sdm_3)	O	O	O	O	O
Pain, numbness or tingling in the hands or feet? (sdm_4)	O	O	O	O	O
Trouble remembering? (sdm_5)	O	O	O	O	O
Nausea or vomiting? (sdm_6)	O	O	O	O	O
Diarrhea or loose bowel movements? (sdm_7)	O	O	O	O	O
Felt sad, down or depressed? (sdm_8)	O	O	O	O	O
Felt nervous or anxious? (sdm_9)	O	O	O	O	O
Difficulty falling or staying asleep? (sdm_10)	O	O	O	O	O

Skin problems, such as rash, dryness or itching? (sdm_11)	O	O	O	O	O
Cough or trouble catching your breath? (sdm_12)	O	O	O	O	O
Headache? (sdm_13)	O	O	O	O	O
Loss of appetite or a change in the taste of food? (sdm_14)	O	O	O	O	O
Bloating, pain or gas in your stomach? (sdm_15)	O	O	O	O	O
Muscle aches or joint pain? (sdm_16)	O	O	O	O	O
Problems with having sex, such as loss of interest or lack of satisfaction? (sdm_17)	O	O	O	O	O
Changes in the way your body looks such as fat deposits or weight gain? (sdm_18)	O	O	O	O	O
Problems with weight loss or wasting? (sdm_19)	O	O	O	O	O

Hair loss or changes in the way your hair looks? (sdm_20)	o	o	o	o	o

End of Block: Symptom Distress Module

Start of Block: Readiness to Quit

ladder_pic

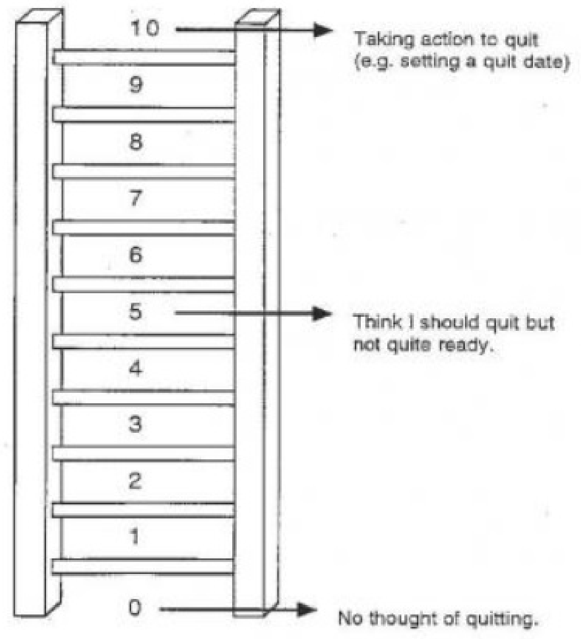

ladder_text Each rung on the ladder represent where various smokers are in their thinking about quitting.

10. I have quit smoking cigarettes and I will never smoke again
9. I have quit smoking cigarettes, but I still worry about slipping back, so I need to keep working on living smoke-free.
8. I still smoke cigarettes, but I have begun to change, like cutting back on the number of cigarettes I smoke. I am ready to set a quit date.

7. I definitely plan to quit smoking cigarettes within the next 30 days.
6. I definitely plan to quit smoking cigarettes within the next 6 months.
5. I often think about quitting smoking cigarettes, but I have no plans to quit.
4. I sometimes think about quitting smoking cigarettes, but I have no plans to quit.
3. I rarely think about quitting smoking cigarettes, but I have no plans to quit.
2. I never think about quitting smoking cigarettes, but I have no plans to quit.
1. I enjoy smoking and have decided not to quit smoking cigarettes for my lifetime. I have no interest in quitting.

---

*

ladder_response What number indicates where you are now?

____

End of Block: Readiness to Quit

Start of Block: Minnesota Withdrawal Scale

withdrawal Please rate yourself on the following symptoms over a period of the last **<u>24 hours</u>**.

	None (0)	Slight (1)	Mild (2)	Moderate (3)	Severe (4)
Angry, irritable, frustrated (2)	O	O	O	O	O
Anxious, nervous (3)	O	O	O	O	O
Depressed mood, sad (4)	O	O	O	O	O
Difficulty concentrating (5)	O	O	O	O	O
Increased appetite, hungry, weight gain (6)	O	O	O	O	O
Insomnia, sleep problems, awakening at night (7)	O	O	O	O	O
Restless (8)	O	O	O	O	O
Desire or craving to smoke (9)	O	O	O	O	O
Constipation (10)	O	O	O	O	O
Coughing (11)	O	O	O	O	O
Decreased pleasure from events (12)	O	O	O	O	O
Dizziness (13)	O	O	O	O	O
Drowsy (14)	O	O	O	O	O

Impatient (15)	O	O	O	O	O
Impulsive (16)	O	O	O	O	O

End of Block: Minnesota Withdrawal Scale

Start of Block: CASE ADHERENCE INDEX & VAS

X→

case1 How often do you feel that you have difficulty taking your HIV medications on time? By "on time" we mean no more than two hours before or two hours after the time your doctor told you to take it.

- O Never (4)
- O Rarely (3)
- O Most of the time (2)
- O All of the time (1)

X→

case2 **On average**, how many days PER WEEK would you say that you missed at least one dose of your HIV medications?

- O Everyday (1)
- O 4-6 days/week (2)
- O 2-3 days/week (3)
- O Once a week (4)
- O Less than once a week (5)
- O Never (6)

case3 When was the last time you missed at least one dose of your HIV medications?

- Within the past week (1)
- 1-2 weeks ago (2)
- 3-4 weeks ago (3)
- Between 1 and 3 months ago (4)
- More than 3 months ago (5)
- Never (6)

Page Break

vas For many people it is difficult to take HIV antiretroviral medications as their doctor prescribes them. The following question asks about your experiences taking HIV antiretroviral medications during the last 4 weeks. Please move the marker on the line below to the point showing your best guess about how much of your HIV antiretroviral medications you have taken in the past 30 days.

0% = you have taken no antiretroviral medications
50% = you have taken 1/2 of your medications
100% = you have taken every dose of your medications

	0	10	20	30	40	50	60	70	80	90	100
Percentage of medication taken in the past 30 days ()											

Page Break

End of Block: CASE ADHERENCE INDEX & VAS

Start of Block: Perceived Stress Scale

pss The questions in this scale ask you about your feelings and thoughts during the **last month**. In each case, you will be asked to indicate by choosing the answer that best describes *how often* you felt or thought a certain way.

	Never (0)	Almost Never (1)	Sometimes (2)	Fairly Often (3)	Very Often (4)
In the last month, how often have you been upset because of something that happened unexpectedly? (pss_1)	O	O	O	O	O
In the last month, how often have you felt that you were unable to control the important things in your life? (pss_2)	O	O	O	O	O
In the last month, how often have you felt nervous or "stressed?" (pss_3)	O	O	O	O	O
In the last month, how often have you felt confident about your ability to handle your personal problems? (pss_4)	O	O	O	O	O
In the last month, how often have you felt that things were going your way? (pss_5)	O	O	O	O	O

In the last month, how often have you found that you could not cope with all the things that you had to do? (pss_6)	O	O	O	O	O
In the last month, how often have you been able to control irritations in your life? (pss_7)	O	O	O	O	O
In the last month, how often have you felt that you were on top of things? (pss_8)	O	O	O	O	O
In the last month, how often have you been angered because of things that were outside of your control? (pss_9)	O	O	O	O	O
In the last month, how often have you felt difficulties were piling up so high that you could not overcome them? (pss_10)	O	O	O	O	O

End of Block: Perceived Stress Scale

Start of Block: CES-D

cesd_instruct Below is a list of the ways you might have felt or behaved.
Please tell me how often you have felt this way during the **past week**.

cesd During the past week...

	Rarely or none of the time (less than 1 day) (0)	Some or a little of the time (1-2 days) (1)	Occasionally or a moderate amount of time (3-4 days) (2)	Most or all of the time (5-7 days) (3)
I was bothered by things that usually don't bother me. (cesd_1)	O	O	O	O
I did not feel like eating; my appetite was poor. (cesd_2)	O	O	O	O
I felt that I could not shake off the blues even with help from my family or friends. (cesd_3)	O	O	O	O
I felt I was just as good as other people. (cesd_4)	O	O	O	O
I had trouble keeping my mind on what I was doing. (cesd_5)	O	O	O	O
I felt depressed. (cesd_6)	O	O	O	O
I felt that everything I did was an effort. (cesd_7)	O	O	O	O
I felt hopeful about the future. (cesd_8)	O	O	O	O
I thought my life had been a failure. (cesd_9)	O	O	O	O
I felt fearful. (cesd_10)	O	O	O	O

My sleep was restless. (cesd_11)	o	o	o	o
I was happy. (cesd_12)	o	o	o	o
I talked less than usual. (cesd_13)	o	o	o	o
I felt lonely. (cesd_14)	o	o	o	o
People were unfriendly. (cesd_15)	o	o	o	o
I enjoyed life. (cesd_16)	o	o	o	o
I had crying spells. (cesd_17)	o	o	o	o
I felt sad. (cesd_18)	o	o	o	o
I felt that people dislike me. (cesd_19)	o	o	o	o
I could not get “going.” (cesd_20)	o	o	o	o

End of Block: CES-D

Start of Block: STAI

X→

stai_1 A number of statements which people have used to describe themselves are given below.

Read each statement and then select the appropriate answer to the right of the statement to indicated how you feel right now, that is, at this moment.

There are no right or wrong answers. Do not spend too much time on any one statement but give the answer which seems to describe your present feelings best.

	Not at all (1)	Somewhat (2)	Moderately so (3)	Very much so (4)
I feel calm (1)	O	O	O	O
I feel secure (2)	O	O	O	O
I am tense (3)	O	O	O	O
I feel strained (4)	O	O	O	O
I feel at ease (5)	O	O	O	O
I feel upset (6)	O	O	O	O
I am presently worrying over possible misfortunes (7)	O	O	O	O
I feel satisfied (8)	O	O	O	O
I feel frightened (9)	O	O	O	O
I feel comfortable (10)	O	O	O	O
I feel self-confident (11)	O	O	O	O
I feel nervous (12)	O	O	O	O
I am jittery (13)	O	O	O	O
I feel indecisive (14)	O	O	O	O
I am relaxed (15)	O	O	O	O

I feel content (16)	o	o	o	o
I am worried (17)	o	o	o	o
I feel confused (18)	o	o	o	o
I feel steady (19)	o	o	o	o
I feel pleasant (20)	o	o	o	o

Page Break

stai_2 A number of statements which people have used to describe themselves are given below.

Read each statement and then select the appropriate answer to the right of the statement to indicated how you ***generally feel***.

There are no right or wrong answers. Do not spend too much time on any one statement but give the answer which seems to describehow you generally feel.

	Almost never (1)	Sometimes (2)	Often (3)	Almost always (4)
I feel pleasant (1)	O	O	O	O
I feel nervous and restless (2)	O	O	O	O
I feel satisfied with myself (3)	O	O	O	O
I wish I could be as happy as others seem to be (4)	O	O	O	O
I feel like a failure (5)	O	O	O	O
I feel rested (6)	O	O	O	O
I am "calm, cool, and collected" (7)	O	O	O	O
I feel that difficulties are piling up so that I cannot overcome them (8)	O	O	O	O
I worry too much over something that really doesn't matter (9)	O	O	O	O
I am happy (10)	O	O	O	O
I have disturbing thoughts (11)	O	O	O	O
I lack self-confidence (12)	O	O	O	O
I feel secure (13)	O	O	O	O

I make decisions easily (14)	o	o	o	o
I feel inadequate (15)	o	o	o	o
I am content (16)	o	o	o	o
Some unimportant thought runs through my mind and bothers me (17)	o	o	o	o
I take disappointments so keenly that I can't put them out of my mind (18)	o	o	o	o
I am a steady person (19)	o	o	o	o
I get in a state of tension or turmoil as I think over my recent concerns and interests (20)	o	o	o	o

End of Block: STAI

Start of Block: Multidimensional Scale of Perceived Social Support

mspss We are interested in how you feel about the following statements. Read each statement carefully. Indicate how you feel about each statement.

	Very strongly disagree (1)	Strongly disagree (2)	Mildly disagreee (3)	Neutral (4)	Mildly agree (5)	Strongly agree (6)	Very strongly agree (7)
There is a special person who is around when I am in need. (1)	O	O	O	O	O	O	O
There is a special person with whom I can share my joys and sorrows. (8)	O	O	O	O	O	O	O
My family really tries to help me. (9)	O	O	O	O	O	O	O
I get the emotional help and support I need from my family. (10)	O	O	O	O	O	O	O
I have a special person who is a real source of comfort to me. (11)	O	O	O	O	O	O	O

My friends really try to help me. (12)	O	O	O	O	O	O	O
I can count on my friends when things go wrong. (13)	O	O	O	O	O	O	O
I can talk about my problems with my family. (14)	O	O	O	O	O	O	O
I have friends with whom I can share my joys and sorrows. (15)	O	O	O	O	O	O	O
There is a special person in my life who cares about my feelings. (2)	O	O	O	O	O	O	O
My family is willing to help me make decisions. (3)	O	O	O	O	O	O	O

I can talk about my problems with my friends. (4)	O	O	O	O	O	O	O

End of Block: Multidimensional Scale of Perceived Social Support

Start of Block: Measuring Change Processes

consciousness Please indicate how often the following statements apply to you.

	Never (1)	Seldom (2)	Occasionally (3)	Frequently (4)	Repeatedly (5)
I recall articles dealing with the problem of quitting smoking. (1)	O	O	O	O	O
I think about information from articles and advertisements on how to stop smoking. (2)	O	O	O	O	O
I recall information people had given me on how to stop smoking. (3)	O	O	O	O	O
I recall information people had personally given me on the benefits of quitting smoking. (4)	O	O	O	O	O

self_liberation Please indicate how often the following statements apply to you.

	Never (1)	Seldom (2)	Occasinoally (3)	Frequently (4)	Repeatedly (5)
I tell myself I can choose to smoke or not. (1)	o	o	o	o	o
I tell myself I am able to quit smoking if I want to. (2)	o	o	o	o	o
I tell myself that if I try hard enough I can keep from smoking. (3)	o	o	o	o	o
I make commitments not to smoke. (4)	o	o	o	o	o

relief Please indicate how often the following statements apply to you.

	Never (1)	Seldom (2)	Occasinoally (3)	Frequently (4)	Repeatedly (5)
Warnings about health hazards of smoking move me emotionally. (1)	O	O	O	O	O
Dramatic portrayals of the evils of smoking affect me emotionally. (2)	O	O	O	O	O
I react emotionally to warnings about smoking cigarettes. (3)	O	O	O	O	O
Remembering studies about illnesses caused by smoking upset me. (4)	O	O	O	O	O

envt_reevaluation Please indicate how often the following statements apply to you.

	Never (1)	Seldom (2)	Occasinoally (3)	Frequently (4)	Repeatedly (5)
I am considering the belief that people quitting smoking will help to improve the world. (1)	o	o	o	o	o
I stop to think that smoking is polluting the environment. (2)	o	o	o	o	o
I consider the view that smoking can be harmful to the environment. (3)	o	o	o	o	o
I am considering the idea that the world could be a better place without my smoking. (4)	o	o	o	o	o

help_relationship Please indicate how often the following statements apply to you.

	Never (1)	Seldom (2)	Occasinoally (3)	Frequently (4)	Repeatedly (5)
Special people in my life accept me the same whether I smoke or not. (help_relationship_1)	o	o	o	o	o
I can be open with at least one special person about my experience with smoking. (help_relationship_2)	o	o	o	o	o
I have someone who listens when I need to talk about my smoking. (help_relationship_3)	o	o	o	o	o
I have someone whom I can count on when I'm having problems with smoking. (help_relationship_4)	o	o	o	o	o

stimulus_control Please indicate how often the following statements apply to you.

	Never (1)	Seldom (2)	Occasinoally (3)	Frequently (4)	Repeatedly (5)
I remove things from my home that remind me of smoking. (1)	O	O	O	O	O
I keep things around my place of work that remind me not to smoke. (2)	O	O	O	O	O
I remove things from my place of work that remind me of smoking. (3)	O	O	O	O	O
I put things around my home that remind me not to smoke. (4)	O	O	O	O	O

countercondition Please indicate how often the following statements apply to you.

	Never (1)	Seldom (2)	Occasinoally (3)	Frequently (4)	Repeatedly (5)
Instead of smoking, I engage in some physical activity. (1)	O	O	O	O	O
I find that doing other things with my hands is a good substitute for smoking. (2)	O	O	O	O	O
When I am tempted to smoke, I think about something else. (3)	O	O	O	O	O
I do something else instead of smoking when I need to relax or deal with tension. (4)	O	O	O	O	O

social_liberation Please indicate how often the following statements apply to you.

	Never (1)	Seldom (2)	Occasinoally (3)	Frequently (4)	Repeatedly (5)
I see "No Smoking" signs in public buildings. (1)	O	O	O	O	O
I notice that public places have sections set aside for smoking. (2)	O	O	O	O	O
I find society changing in ways that make it easier for the nonsmoker. (3)	O	O	O	O	O
I notice that nonsmokers are asserting their rights. (5)	O	O	O	O	O

self_reevaluation Please indicate how often the following statements apply to you.

	Never (1)	Seldom (2)	Occasinoally (3)	Frequently (4)	Repeatedly (5)
My dependency on cigarettes makes me feel disappointment in myself. (1)	o	o	o	o	o
I get upset when I think about my smoking. (2)	o	o	o	o	o
I reassess the fact that being content with myself includes changing the smoking habit. (3)	o	o	o	o	o
I consciously struggle with the issue that smoking contradicts my view of myself as a caring and responsible person. (4)	o	o	o	o	o

reinforce_manage Please indicate how often the following statements apply to you.

	Never (1)	Seldom (2)	Occasinoally (3)	Frequently (4)	Repeatedly (5)
I can expect to be rewarded by others if I don't smoke. (1)	o	o	o	o	o
I am rewarded by others if I don't smoke. (2)	o	o	o	o	o
Other people in my daily life try to make me feel good when I don't smoke. (3)	o	o	o	o	o
I reward myself when I don't smoke. (4)	o	o	o	o	o

Page Break

End of Block: Measuring Change Processes

Start of Block: TOHFLA

tohfla_instructions1 **Here are some medical instructions that you or anybody might see around the hospital. These instructions are in sentences that have some of the words missing. Where a word is missing, a blank line is drawn, and 4 possible words that could go in the blank appear just below it. Figure out which of those 4 words should go in the blank, which word makes the sentence make sense. When you think you know which one it is, select the word by clicking on the word or the button next to the word for each blank space.**

tohfla_instructions2 PASSAGE A: X-RAY PREPARATION

---

tohfla_instructions3 Your doctor has sent you to have a **__(1)__** X-ray.
a. stomach
b. diabetes
c. stitches
d. germs

X→

tohfla_a1 Select the word that goes in blank (1) above.

- O stomach (1)
- O diabetes (2)
- O stitches (3)
- O germs (4)

---

tohfla_instructions4 You must have an **__(2)__** stomach when you come for **__(3)__** .
a. asthma   a. is   b. empty   b. am   c. incest   c. if   d. anemia   d. it

---

X→

tohfla_a2 Select the word that goes in blank (2) above.

- O asthma (1)
- O empty (2)
- O incest (3)
- O anemia (4)

tohfla_a3 Select the word that goes in blank (3) above.

- O is (1)
- O am (2)
- O if (3)
- O it (4)

tohfla_instructions5 The X-ray will ___**(4)**___ from 1 to 3 ___**(5)**___ to do.

a. take
a. beds

b. view
b. brains

c. talk
c. hours

d. look
d. diets

tohfla_a4 Select the word that goes in blank (4) above.

- O take (1)
- O view (2)
- O talk (3)
- O look (4)

tohfla_a5 Select the word that goes in blank (5) above.

- O beds (1)
- O brains (2)
- O hours (3)
- O diets (4)

Page Break

tohfla_instructions6 THE DAY BEFORE THE X-RAY

tohfla_instructions7 For supper have only a ___**(6)**___ snack of fruit, ___**(7)**___ and jelly, with coffee or tea.

a. little
a. toes
b. broth
b. throat
c. attack
c. toast
d. nausea
d. thigh

tohfla_a6 Select the word that goes in blank (6) above.

- O little (1)
- O broth (2)
- O attack (3)
- O nausea (4)

---

tohfla_a7 Select the word that goes in blank (7) above.

- O toes (1)
- O throat (2)
- O toast (3)
- O thigh (4)

---

tohfla_instructions8 After __**(8)**__ , you must not __**(9)**__ or drink anything at __**(10)**__ until after you have __**(11)**__ the X-ray.

a. minute
a. easy a. ill a.
are b. midnight
b. ate b. all b. has
c. during c. drank
c. each c. had
d. before d. eat d. any d. was

---

tohfla_a8 Select the word that goes in blank (8) above.

- minute (1)
- midnight (2)
- during (3)
- before (4)

---

tohfla_a9 Select the word that goes in blank (9) above.

- easy (1)
- ate (2)
- drank (3)
- eat (4)

---

tohfla_a10 Select the word that goes in blank (10) above.

- ill (1)
- all (2)
- each (3)
- any (4)

---

tohfla_a11 Select the word that goes in blank (11) above.

- O are (1)
- O has (2)
- O had (3)
- O was (4)

Page Break

tohfla_instructions9 THE DAY OF THE X-RAY

tohfla_instruct10 Do not eat __**(12)**__ .

a. appointment
b. walk-in
c. breakfast
d. clinic

X→

tohfla_a12 Select the word that goes in blank (12) above.

- O appointment (1)
- O walk-in (2)
- O breakfast (3)
- O clinic (4)

tohfla_instruct11 Do not ___**(13)**___ , even ___**(14)**___ .
a. drive a. heart
b. drink b. breath
c. dress
c. water d.
dose d. cancer

---

tohfla_a13 Select the word that goes in blank (13) above.

- O drive (1)
- O drink (2)
- O dress (3)
- O dose (4)

---

tohfla_a14 Select the word that goes in blank (14) above.

- O heart (1)
- O breath (2)
- O water (3)
- O cancer (4)

---

tohfla_instruct12 If you have any ___(15)___ , call the X-ray ___(16)___ at 616-4500.
a.
answers a. Department
b. exercises b. Sprain
c. tracts c. Pharmacy
d. questions d.
Toothache

tohfla_a15 Select the word that goes in blank (15) above.

- O answers (1)
- O exercises (2)
- O tracts (3)
- O questions (4)

tohfla_a16 Select the word that goes in blank (16) above.

- O Department (1)
- O Sprain (2)
- O Pharmacy (3)
- O Toothache (4)

Page Break

tohfla_instruct13 PASSAGE B: MEDICAID RIGHTS AND RESPONSBILITIES

tohfla_instruct14 I agree to give correct information to ___**(1)**___ if I can receive Medicaid.

a. hair
b. salt
c. see
d. ache

---

tohfla_b17 Select the word that goes in blank (1) above.

- O hair (1)
- O salt (2)
- O see (3)
- O ache (4)

---

tohfla_instruct15 I ___**(2)**___ to provide the county information to ___**(3)**___ any statements given in this ___**(4)**___

a.
agree a. hide
a. emphysema
b. probe b. risk b. application
c. send
c. discharge c. gallbladder
d. gain d.
prove d. relationship

and hereby give permission to the ___**(5)**___ to get such proof.

a. inflammation
b. religion
c. iron
d. county

---

tohfla_b18 Select the word that goes in blank (2) above.

- O agree (1)
- O probe (2)
- O send (3)
- O gain (4)

tohfla_b19 Select the word that goes in blank (3) above.

- O hide (1)
- O risk (2)
- O discharge (3)
- O prove (4)

tohfla_b20 Select the word that goes in blank (4) above.

- O emphysema (1)
- O application (2)
- O gallbladder (3)
- O relationship (4)

tohfla_b21 Select the word that goes in blank (5) above.

- O inflammation (1)
- O religion (2)
- O iron (3)
- O county (4)

---

tohfla_instruct16 I ___(6)___ that for Medicaid I must report any ___(7)___ in my circumstances within ___(8)___ (10) days

a.
investigate
a. changes
a. three
b. entertain
b. hormones
b. one
c. understand
c. antacids
c. five
d. establish
d. charges
d. ten

of becoming ___(9)___ of the change.

a. award
b. aware
c. away
d. await

---

tohfla_b22 Select the word that goes in blank (6) above.

- O investigate (1)
- O entertain (2)
- O understand (3)
- O establish (4)

---

tohfla_b23 Select the word that goes in blank (7) above.

- O changes (1)
- O hormones (2)
- O antacids (3)
- O charges (4)

---

tohfla_b24 Select the word that goes in blank (8) above.

- O three (1)
- O one (2)
- O five (3)
- O ten (4)

---

tohfla_b25 Select the word that goes in blank (9) above.

- O award (1)
- O aware (2)
- O away (3)
- O await (4)

---

tohfla_instruct17 I understand ___(10)___ if I DO NOT like the ___(11)___ made on my case, I have the ___(12)___ to a fair hearing.

a. thus
a.
marital
a. bright
b. this
b. occupation
b. left
c. that
c. adult
c. wrong
d. than
d. decision
d. right

---

tohfla_b26 Select the word that goes in blank (10) above.

O thus (1)

O this (2)

O that (3)

O than (4)

---

tohfla_b27 Select the word that goes in blank (11) above.

O marital (1)

O occupation (2)

O adult (3)

O decision (4)

---

tohfla_b28 Select the word that goes in blank (12) above.

- O bright (1)
- O left (2)
- O wrong (3)
- O right (4)

---

tohfla_instruct18 I can ___(13)___ a hearing by writing or ___(14)___ the county where I applied.
a. request a.
counting b. refuse
b. reading
c. fail c. calling
d. mend d. smelling

---

tohfla_b29 Select the word that goes in blank (13) above.

- O request (1)
- O refuse (2)
- O fail (3)
- O mend (4)

---

tohfla_b30 Select the word that goes in blank (14) above.

- counting (1)
- reading (2)
- calling (3)
- smelling (4)

---

tohfla_instruct19 If you ___(15)___ TANF for any family ___(16)___, you will have to ___(17)___ a different application form. a.
wash a. member a.
relax b. want
b. history b. break
c. cover c. weight c.
inhale d. tape
d. seatbelt d. sign

---

tohfla_b31 Select the word that goes in blank (15) above.

- wash (1)
- want (2)
- cover (3)
- tape (4)

---

tohfla_b32 Select the word that goes in blank (16) above.

- O member (1)
- O history (2)
- O weight (3)
- O seatbelt (4)

tohfla_b33 Select the word that goes in blank (17) above.

- O relax (1)
- O break (2)
- O inhale (3)
- O sign (4)

tohfla_instruct20 __(18)__ , we will use the __(19)__ on this form to determine your __(20)__ .
a. Since a. lung a. hypoglycemia
b. Whether b. date b. eligibility
c. However c. meal c. osteoporosis
d. Because d. pelvic d. schizophrenia

tohfla_b34 Select the word that goes in blank (18) above.

- O Since (1)
- O Whether (2)
- O However (3)
- O Because (4)

---

tohfla_b35 Select the word that goes in blank (19) above.

- O lung (1)
- O date (2)
- O meal (3)
- O pelvic (4)

---

tohfla_b36 Select the word that goes in blank (20) above.

- O hypoglycemia (1)
- O eligibility (2)
- O osteoporosis (3)
- O schizophrenia (4)

Page Break

End of Block: TOHFLA

Start of Block: COVID-19 v2

X→

covid_dx_v2 In the last 3 months (since ${e://Field/covid_todayminus90}), have you been diagnosed with COVID-19 by a doctor or another health care provider?

- O Yes (1)
- O No (2)
- O I don't know (999)
- O I was diagnosed with COVID-19 prior to ${e://Field/covid_todayminus90} (888)

Display This Question:

If covid_dx_v2 = 1

Or covid_dx_v2 = 888

covid_hosp_v2 Were you hospitalized due to your COVID-19 diagnosis?

- O Yes (1)
- O No (2)

X→

covid_dx_others_v2 In the last 3 months (since ${e://Field/covid_todayminus90}), have any of your close friends or family members been diagnosed with COVID-19 by a doctor or another health care provider?

- O Yes (1)
- O No (2)
- O I don't know (999)

X→

covid_concern_v2 Overall, how concerned are you about the COVID-19 pandemic?

- O Not at all concerned (0)
- O A little concerned (1)
- O Somewhat concerned (2)
- O Very concerned (3)

Page Break

X→

covid_order_v2 Due to the COVID-19 pandemic, several cities, counties, and states have had one or more “Shelter in Place/Stay at Home” orders or advisories. Additionally, some people have voluntarily sheltered in place/stayed at home for different periods of time. By this we mean that you stayed at home and only left home to get food, medicine, heath care services, or for work if you are an essential worker. In the last 3 months (since ${e://Field/covid_todayminus90}), have you sheltered-in-place/stayed-at-home either voluntarily or due to a city, county, or state mandate/advisory due to the COVID-19 pandemic?

- O Yes (1)
- O No (2)

Skip To: covid_medcare_v2 If covid_order_v2 = 2

X→

covid_order_desc_v2 Which of the following best describes your voluntary or mandated shelter-in-place/stay-at-home in the last 3 months, since ${e://Field/covid_todayminus90}?

- O Have continued to "Shelter in Place/Stay at Home" and have been since before ${e://Field/covid_todayminus90} (1)
- O Stopped a “Shelter in Place/Stay at Home” that started prior to ${e://Field/covid_todayminus90} and have not been sheltering/staying at home since (2)
- O Stopped a “Shelter in Place/Stay at Home” that started prior to ${e://Field/covid_todayminus90} and have since engaged in another “Shelter in Place/Stay at Home” (3)
- O Have only started a “Shelter in Place/Stay at Home” (4)

Display This Question:

If covid_order_desc_v2 = 2

Or covid_order_desc_v2 = 3

covid_order_stop_v2 You said you stopped a “Shelter in Place/Stay at Home” that started prior to ${e://Field/covid_todayminus90}. On approximately, what date did you stop?

- O End Date (MM/DD/YYYY) (1) ____

Display This Question:

If covid_order_desc_v2 = 3

Or covid_order_desc_v2 = 4

covid_order_type_v2 You said you have begun a “Shelter in Place/Stay at Home” in the last 3 months due to the COVID-19 pandemic. Is this a city, county, state or other locale mandated order/advisory, or are you voluntarily sheltering in place?

- O City, County, State or Locale Mandated/Advisory “Shelter in Place/Stay at Home” (1)
- O Voluntary “Shelter in Place/Stay at Home” (2)

Display This Question:

If covid_order_desc_v2 = 3

Or covid_order_desc_v2 = 4

And If

covid_order_type_v2 = 1

covid_order_fllw_v2 Which government-level **Shelter in Place/Stay at Home Orders** are you following? *(Please select all that apply.)*

▢ City (please specify) (1) ____

▢ County (please specify) (2) ____

▢ State (please specify) (3) ____

Display This Question:

If covid_order_desc_v2 = 3

Or covid_order_desc_v2 = 4

quar_actv_v2 Are you still sheltering in place/staying at home due to COVID-19?

O Yes (1)

O No (2)

Display This Question:

If covid_order_desc_v2 = 3

Or covid_order_desc_v2 = 4

quar_dates_v2 On approximately what date did your "**Shelter in Place/Stay at Home**" start (and end, if applicable) in the last 3 months? (Example: 05/14/2020)

O Start Date (MM/DD/YYYY) (1) ____

Display This Choice:

If quar_actv_v2 = 2

O End Date (MM/DD/YYYY) (2) ____

X→

covid_medcare_v2 In the last 3 months (since ${e://Field/covid_todayminus90}), how concerned have you been about your ability to access medical care during the COVID-19 outbreak?

O Not at all concerned (0)

O A little concerned (1)

O Somewhat concerned (2)

O Very concerned (3)

X→

art_covid_v2 In the last 3 months (since ${e://Field/covid_todayminus90}), have you used ART (HIV medication)?

O Yes (1)

O No (0)

Skip To: covid_art_conc_v2 If art_covid_v2 = 0

X→

covid_art_change_v2 In the last 3 months (since ${e://Field/covid_todayminus90}) during the COVID-19 outbreak, have you changed how you take ART (HIV medication)?

- O No, my ART use hasn't changed (0)
- O Yes, I stopped taking ART as often as I did before (1)
- O Yes, my ART use changed in some ways but did not stop completely (please specify) (2) ____

Skip To: art_prov_contact_v2 If covid_art_change_v2 = 0

X→

covid_stop_art_v2 Why did you stop taking ART (HIV medication) as often or completely?

- O I don't think I need it anymore (please specify) (1) ____
- O I don't have access to it anymore (2)
- O I stopped for some other reason (please specify) (3) ____

Skip To: End of Block If covid_stop_art_v2 = 1

Display This Question:

If covid_stop_art_v2 = 2

covid_art_noacc_v2 Why do you not have access to ART (HIV medication) anymore?

- O I don't have health insurance anymore (1)
- O My provider can't prescribe or refill my prescription (2)
- O I can't complete routine testing/labs required for my prescription (3)
- O Some other reason (please specify) (4) ____

art_prov_contact_v2 In the last 3 months (since ${e://Field/covid_todayminus90}), have you reached out to your provider for an ART (HIV medication) refill?

- Yes, I have (1)
- No, I have not (0)
- No, I have more than a 90-day supply (999)

Display This Question:

If art_prov_contact_v2 = 1

covid_prov_offer_v2 Did your provider offer any of the following services when you reached out about an ART (HIV medication) refill?

- My provider offered a refill without the quarterly visit (1)
- My provider offered a telemedicine (e.g., video call) appointment to refill my prescription (2)
- My provider offered an office-based appointment to refill my prescription (3)
- My provider offered something else (please specify) (4) ____

covid_art_conc_v2 In the last 3 months (since ${e://Field/covid_todayminus90}), how concerned have you been about accessing ART (HIV medication) during the COVID-19 outbreak?

- Not at all concerned (0)
- A little concerned (1)
- Somewhat concerned (2)
- Very concerned (3)

End of Block: COVID-19 v2
