# Supplementary material for: A Smoking Cessation Mobile App for Persons Living With HIV: Preliminary Efficacy and Feasibility Study
Source: JMIR Form Res. 2022 Aug 18;6(8):e28626. doi: 10.2196/28626 (PMC9437787; doi:10.2196/28626)
Supplement: Multimedia Appendix 5 [file formative_v6i8e28626_app5.pdf]

Smoking Cessation Pilot FU

Start of Block: Introduction

qs_id
Smoking Cessation Pilot FU

User ID

____

arm Please select the study arm

- L (4)
- C (5)

Visit_Type Please select the visit type

- In-Person (1)
- Remote (2)

Display This Question:

If Visit_Type = 2

12wk_PPM What was your reading when you complete the breath test with the iCO Smokerlyzer?

- ○ 0-6 ppm (1)
- ○ 7-10 ppm (2)
- ○ 11-15 ppm (3)
- ○ 16-20 ppm (4)
- ○ 21-25 ppm (5)
- ○ 26-30 ppm (6)
- ○ 31 Plus ppm (7)
- ○ I did not complete a breath test (8)

End of Block: Introduction

Start of Block: Demographics

work What is your current employment status? (Check all that apply)

- ▢ Working full-time (1)
- ▢ Working part-time (including seasonal, work-study, etc.) (2)
- ▢ Working off the books (not reported as taxable income) (3)
- ▢ Unemployed, looking for work (4)
- ▢ Unemployed, not looking for work (5)
- ▢ Retired (6)
- ▢ Student (7)
- ▢ Disabled (8)

---

healthinsur Do you currently have health insurance? (Check all that apply)

- ☐ Yes - Through my job (1)
- ☐ Yes - Through someone else's job (2)
- ☐ Yes - Through a health exchange (Affordable Care Act) (3)
- ☐ Yes - Paid for by me or another person (4)
- ☐ Yes - Medicaid/Medicare (5)
- ☐ Yes - ADAP (6)
- ☐ Yes - Veteran's Association (7)
- ☐ Other (please specify): (8) ____
- ☐ ⊗No (9)
- ☐ ⊗I don't know (10)

Page Break

meds Are you **currently** taking any medications prescribed by your doctors? (Check all that apply)

- ☐ Antibiotics (e.g., Bactrim) (1)
- ☐ Anxiety medication (2)
- ☐ Bipolar medication (3)
- ☐ Blood pressure medication (4)
- ☐ Cancer medication or treatment (5)
- ☐ Cholesterol medication (6)
- ☐ Contraceptive pill (7)
- ☐ Depression medication (8)
- ☐ Diabetes medication (9)
- ☐ Erection medication (e.g., Viagra) (10)
- ☐ Heart medication (11)
- ☐ Herpes medication (12)
- ☐ Hormone replacement therapy (e.g., Premarin, Estrogel) (13)
- ☐ Schizophrenia medication (14)
- ☐ Seizure medication (15)
- ☐ Steroids (16)
- ☐ Testosterone (17)

☐ Other (please specify): (18) ____

☐ ⊗None of the above (0)

Page Break

healthutil_1a A healthcare provider could be a general doctor, a specialist doctor, a nurse practitioner, a physician assistant, a nurse, or anyone else you would see for health care.

People visit healthcare providers for many reasons including illness, injury, chronic health conditions, mental health treatment or therapy, substance use treatment, and other types of care. People also see their healthcare providers for regular visits such as check-ups in order to assess overall health, not usually prompted by a specific illness or complaint. People also routinely see their healthcare providers to get their medication prescriptions filled.

In the **last 30 days**, how many times have you visited a healthcare provider to get care for yourself for any reason (including visits to the hospital, ER, doctor's office, or clinic)?

____

Q377 Thinking of the times you have seen a healthcare provider in the **last 30 days,** how many of these visits were for an illness, sickness, or concern and NOT for a check-up or routine care?

____

Page Break

art Are you **currently** taking antiretroviral medications to treat your HIV infection?

- Yes (1)
- No (0)

End of Block: Demographics

Start of Block: Tobacco Use History

7-day pt prev In the **past 7 days**, have you smoked cigarettes or used other forms of tobacco?

- Yes (1)
- No (2)

Skip To: nrt If 7-day pt prev = 2

tobaccotypesmoke What forms of tobacco have you smoked in the past 7 days? Please select **all** products.

- ☐ Cigarettes (1)
- ☐ Cigars (2)
- ☐ Cigarillos (e.g. Black and Mild) (3)
- ☐ Little Cigars (e.g. Winchester) (4)
- ☐ Pipe filled with tobacco (5)
- ☐ Hookah (6)
- ☐ Bidis (7)
- ☐ Clove Cigarettes (8)
- ☐ Marijuana/Tobacco combinations (e.g. spliff, blunt) (9)
- ☐ Other (please specify): (10) ____

tobaccotypesmokeless What other **smokeless** forms of tobacco have you used in the past 7 days? Please select **all** products.

- ☐ Chewing Tobacco (e.g. Redman) (1)
- ☐ Snuff (e.g. Skoal) (2)
- ☐ Snus (e.g. Camel Snus) (3)
- ☐ Dissolvables (e.g. Camel Orbs) (4)
- ☐ Other (please specify): (5) ____
- ☐ ⊗I did not use any smokeless tobacco products in the last 7 days (6)

X→

nrt What type of nicotine replacement therapy have you used/been using during the course of the study?
Select all that apply.

- ☐ Patch (1)
- ☐ Inhaler (2)
- ☐ Nasal spray (6)
- ☐ Lozenge (3)
- ☐ Gum (4)
- ☐ Tablet (7)
- ☐ Other (please specify): (5) ____

End of Block: Tobacco Use History

Start of Block: PROMIS-29

promis_pf **Physical Function**

	Without any difficulty (5)	With a little difficulty (4)	With some difficulty (3)	With much difficulty (2)	Unable to do (1)
Are you able to do chores such as vacuuming or yard work? (1)	○	○	○	○	○
Are you able to go up and down stairs at a normal pace? (2)	○	○	○	○	○
Are you able to go for a walk of at least 15 minutes? (3)	○	○	○	○	○
Are you able to run errands and shop? (4)	○	○	○	○	○

promis_anx
Anxiety

In the past 7 days…

	Never (1)	Rarely (2)	Sometimes (3)	Often (4)	Always (5)
I felt fearful. (1)	○	○	○	○	○
I found it hard to focus on anything other than my anxiety. (2)	○	○	○	○	○
My worries overwhelmed me. (3)	○	○	○	○	○
I felt uneasy. (4)	○	○	○	○	○

---

promis_dep

Depression

In the past 7 days…

	Never (1)	Rarely (2)	Sometimes (3)	Often (4)	Always (5)
I felt worthless. (1)	○	○	○	○	○
I felt helpless. (2)	○	○	○	○	○
I felt depressed. (3)	○	○	○	○	○
I felt hopeless. (4)	○	○	○	○	○

---

promis_fat
<u>Fatigue</u>

	Not at all (1)	A little bit (2)	Somewhat (3)	Quite a bit (4)	Very much (5)
**During the past 7 days…** I feel fatigued. (promis_fat_1)	○	○	○	○	○
I have trouble <u>starting</u> things because I am tired. (promis_fat_2)	○	○	○	○	○
**In the past 7 days…** How run-down did you feel on average? (promis_fat_3)	○	○	○	○	○
How fatigued were you on average? (promis_fat_4)	○	○	○	○	○

X→

promis_sd1 **<u>Sleep Disturbance</u>**

In the past 7 days…

	Very poor (5)	Poor (4)	Fair (3)	Good (2)	Very good (1)
My sleep quality was... (promis_sd_1)	○	○	○	○	○

X→

promis_sd2 **In the past 7 days…**

	Very much (1)	Quite a bit (2)	Somewhat (3)	A little bit (4)	Not at all (5)
My sleep was refreshing. (1)	○	○	○	○	○

promis_sd3-4 **In the past 7 days…**

	Not at all (1)	A little bit (2)	Somewhat (3)	Quite a bit (4)	Very much (5)
I had a problem with my sleep. (promis_sd_3)	○	○	○	○	○
I had difficulty falling asleep. (promis_sd_4)	○	○	○	○	○

promis_ssr
Satisfaction with Social Role

In the past 7 days…

	Not at all (1)	A little bit (2)	Somewhat (3)	Quite a bit (4)	Very much (5)
I am satisfied with how much work I can do (include work at home). (1)	○	○	○	○	○
I am satisfied with my ability to work (include work at home). (2)	○	○	○	○	○
I am satisfied with my ability to do regular personal and household responsibilities. (3)	○	○	○	○	○
I am satisfied with my ability to perform my daily routines. (4)	○	○	○	○	○

promis_pi **<u>Pain Interference</u>**
In the past 7 days…

	Not at all (1)	A little bit (2)	Somewhat (3)	Quite a bit (4)	Very much (5)
How much did pain interfere with your day to day activities? (1)	○	○	○	○	○
How much did pain interfere with work around the home? (2)	○	○	○	○	○
How much did pain interfere with your ability to participate in social activities? (3)	○	○	○	○	○
How much did pain interfere with your household chores? (4)	○	○	○	○	○

promis_painint **Pain Intensity** **In the past 7 days…**
How would you rate your pain on average?

- ○ **No pain**0 (0)
- ○ 1 (1)
- ○ 2 (2)
- ○ 3 (3)
- ○ 4 (4)
- ○ 5 (5)
- ○ 6 (6)
- ○ 7 (7)
- ○ 8 (8)
- ○ 9 (9)
- ○ **Worstimaginable pain**10 (10)

End of Block: PROMIS-29

Start of Block: Symptom Distress Module

X→

sdm During the **past four weeks**, did you have.............?

	**No**, I did NOT HAVE THIS SYMPTOM (0)	Yes, I have this symptom. **It did NOT bother me** (1)	Yes, I have this symptom. **It bothered me a little** (2)	Yes, I have this symptom. **It bothered me** (3)	Yes, I have this symptom. **It bothered me a lot** (4)
Fatigue or loss of energy? (sdm_1)	○	○	○	○	○
Fevers, chills or sweats? (sdm_2)	○	○	○	○	○
Feeling dizzy or lightheaded? (sdm_3)	○	○	○	○	○
Pain, numbness or tingling in the hands or feet? (sdm_4)	○	○	○	○	○
Trouble remembering? (sdm_5)	○	○	○	○	○
Nausea or vomiting? (sdm_6)	○	○	○	○	○
Diarrhea or loose bowel movements? (sdm_7)	○	○	○	○	○
Felt sad, down or depressed? (sdm_8)	○	○	○	○	○
Felt nervous or anxious? (sdm_9)	○	○	○	○	○
Difficulty falling or staying asleep? (sdm_10)	○	○	○	○	○

Skin problems, such as rash, dryness or itching? (sdm_11)	○	○	○	○	○
Cough or trouble catching your breath? (sdm_12)	○	○	○	○	○
Headache? (sdm_13)	○	○	○	○	○
Loss of appetite or a change in the taste of food? (sdm_14)	○	○	○	○	○
Bloating, pain or gas in your stomach? (sdm_15)	○	○	○	○	○
Muscle aches or joint pain? (sdm_16)	○	○	○	○	○
Problems with having sex, such as loss of interest or lack of satisfaction? (sdm_17)	○	○	○	○	○
Changes in the way your body looks such as fat deposits or weight gain? (sdm_18)	○	○	○	○	○
Problems with weight loss or wasting? (sdm_19)	○	○	○	○	○

Hair loss or changes in the way your hair looks? (sdm_20)	○	○	○	○	○

End of Block: Symptom Distress Module

Start of Block: Readiness to Quit

ladder_pic

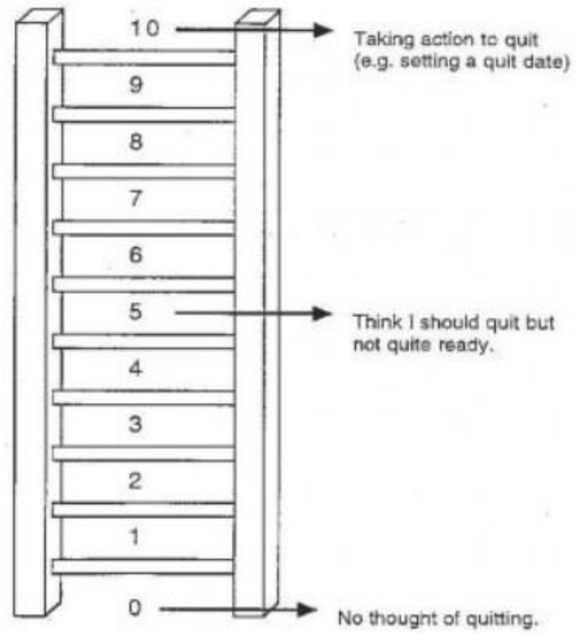

ladder_text Each rung on the ladder represent where various smokers are in their thinking about quitting.

10. I have quit smoking cigarettes and I will never smoke again
9. I have quit smoking cigarettes, but I still worry about slipping back, so I need to keep working on living smoke-free.
8. I still smoke cigarettes, but I have begun to change, like cutting back on the number of cigarettes I smoke. I am ready to set a quit date.

7. I definitely plan to quit smoking cigarettes within the next 30 days.
6. I definitely plan to quit smoking cigarettes within the next 6 months.
5. I often think about quitting smoking cigarettes, but I have no plans to quit.
4. I sometimes think about quitting smoking cigarettes, but I have no plans to quit.
3. I rarely think about quitting smoking cigarettes, but I have no plans to quit.
2. I never think about quitting smoking cigarettes, but I have no plans to quit.
1. I enjoy smoking and have decided not to quit smoking cigarettes for my lifetime. I have no interest in quitting.

---

*

ladder_response What number indicates where you are now?

____

End of Block: Readiness to Quit

Start of Block: Minnesota Withdrawal Scale

withdrawal Please rate yourself on the following symptoms over a period of the last **24 hours**.

	None (0)	Slight (1)	Mild (2)	Moderate (3)	Severe (4)
Angry, irritable, frustrated (2)	○	○	○	○	○
Anxious, nervous (3)	○	○	○	○	○
Depressed mood, sad (4)	○	○	○	○	○
Difficulty concentrating (5)	○	○	○	○	○
Increased appetite, hungry, weight gain (6)	○	○	○	○	○
Insomnia, sleep problems, awakening at night (7)	○	○	○	○	○
Restless (8)	○	○	○	○	○
Desire or craving to smoke (9)	○	○	○	○	○
Constipation (10)	○	○	○	○	○
Coughing (11)	○	○	○	○	○
Decreased pleasure from events (12)	○	○	○	○	○
Dizziness (13)	○	○	○	○	○
Drowsy (14)	○	○	○	○	○

Impatient (15)	○	○	○	○	○
Impulsive (16)	○	○	○	○	○

End of Block: Minnesota Withdrawal Scale

Start of Block: VAS

vas For many people it is difficult to take HIV antiretroviral medications as their doctor prescribes them. The following question asks about your experiences taking HIV antiretroviral medications during the last 4 weeks. Please move the marker on the line below to the point showing your best guess about how much of your HIV antiretroviral medications you have taken in the past 30 days.

0% = you have taken no antiretroviral medications
50% = you have taken 1/2 of your medications
100% = you have taken every dose of your medications

	0 10 20 30 40 50 60 70 80 90 100
Percentage of medication taken in the past 30 days ()	

trap Please select the number 4 below. This helps prevent automated programs from abusing the study.

- 6 (1)
- 5 (2)
- 4 (3)
- 3 (4)
- 2 (5)
- 1 (6)

End of Block: VAS

Start of Block: Perceived Stress Scale

pss The questions in this scale ask you about your feelings and thoughts during the **last month**. In each case, you will be asked to indicate by choosing the answer that best describes *how often* you felt or thought a certain way.

	Never (0)	Almost Never (1)	Sometimes (2)	Fairly Often (3)	Very Often (4)
In the last month, how often have you been upset because of something that happened unexpectedly? (1)	○	○	○	○	○
In the last month, how often have you felt that you were unable to control the important things in your life? (2)	○	○	○	○	○
In the last month, how often have you felt nervous or "stressed?" (3)	○	○	○	○	○
In the last month, how often have you felt confident about your ability to handle your personal problems? (4)	○	○	○	○	○
In the last month, how often have you felt that things were going your way? (5)	○	○	○	○	○

In the last month, how often have you found that you could not cope with all the things that you had to do? (6)	○	○	○	○	○
In the last month, how often have you been able to control irritations in your life? (7)	○	○	○	○	○
In the last month, how often have you felt that you were on top of things? (8)	○	○	○	○	○
In the last month, how often have you been angered because of things that were outside of your control? (9)	○	○	○	○	○
In the last month, how often have you felt difficulties were piling up so high that you could not overcome them? (10)	○	○	○	○	○

End of Block: Perceived Stress Scale

Start of Block: CES-D

cesd_instruct Below is a list of the ways you might have felt or behaved.
Please tell me how often you have felt this way during the **past week**.

cesd During the past week...

	Rarely or none of the time (less than 1 day) (0)	Some or a little of the time (1-2 days) (1)	Occasionally or a moderate amount of time (3-4 days) (2)	Most or all of the time (5-7 days) (3)
I was bothered by things that usually don't bother me. (1)	○	○	○	○
I did not feel like eating; my appetite was poor. (2)	○	○	○	○
I felt that I could not shake off the blues even with help from my family or friends. (3)	○	○	○	○
I felt I was just as good as other people. (4)	○	○	○	○
I had trouble keeping my mind on what I was doing. (5)	○	○	○	○
I felt depressed. (6)	○	○	○	○
I felt that everything I did was an effort. (7)	○	○	○	○
I felt hopeful about the future. (8)	○	○	○	○
I thought my life had been a failure. (9)	○	○	○	○
I felt fearful. (10)	○	○	○	○
My sleep was restless. (11)	○	○	○	○

I was happy. (12)	○	○	○	○
I talked less than usual. (13)	○	○	○	○
I felt lonely. (14)	○	○	○	○
People were unfriendly. (15)	○	○	○	○
I enjoyed life. (16)	○	○	○	○
I had crying spells. (17)	○	○	○	○
I felt sad. (18)	○	○	○	○
I felt that people dislike me. (19)	○	○	○	○
I could not get “going.” (20)	○	○	○	○

End of Block: CES-D

Start of Block: STAI

stai_1 A number of statements which people have used to describe themselves are given below.

Read each statement and then select the appropriate answer to the right of the statement to indicated how you feel right now, that is, at this moment.

There are no right or wrong answers. Do not spend too much time on any one statement but give the answer which seems to describe your present feelings best.

	Not at all (1)	Somewhat (2)	Moderately so (3)	Very much so (4)
I feel calm (1)	○	○	○	○
I feel secure (2)	○	○	○	○
I am tense (3)	○	○	○	○
I feel strained (4)	○	○	○	○
I feel at ease (5)	○	○	○	○
I feel upset (6)	○	○	○	○
I am presently worrying over possible misfortunes (7)	○	○	○	○
I feel satisfied (8)	○	○	○	○
I feel frightened (9)	○	○	○	○
I feel comfortable (10)	○	○	○	○
I feel self-confident (11)	○	○	○	○
I feel nervous (12)	○	○	○	○
I am jittery (13)	○	○	○	○
I feel indecisive (14)	○	○	○	○
I am relaced (15)	○	○	○	○

I feel content (16)	○	○	○	○
I am worried (17)	○	○	○	○
I feel confused (18)	○	○	○	○
I feel steady (19)	○	○	○	○
I feel pleasant (20)	○	○	○	○

---

stai_2 A number of statements which people have used to describe themselves are given below.

Read each statement and then select the appropriate answer to the right of the statement to indicated how you ***generally feel***.

There are no right or wrong answers. Do not spend too much time on any one statement but give the answer which seems to describehow you generally feel.

	Almost never (1)	Sometimes (2)	Often (3)	Almost always (4)
I feel pleasant (1)	○	○	○	○
I feel nervous and restless (2)	○	○	○	○
I feel satisfied with myself (3)	○	○	○	○
I wish I could be as happy as others seem to be (4)	○	○	○	○
I feel like a failure (5)	○	○	○	○
I feel rested (6)	○	○	○	○
I am "calm, cool, and collected" (7)	○	○	○	○
I feel that difficulties are piling up so that I cannot overcome them (8)	○	○	○	○
I worry too much over something that really doesn't matter (9)	○	○	○	○
I am happy (10)	○	○	○	○
I have disturbing thoughts (11)	○	○	○	○
I lack self-confidence (12)	○	○	○	○
I feel secure (13)	○	○	○	○

I make decisions easily (14)	○	○	○	○
I feel inadequate (15)	○	○	○	○
I am content (16)	○	○	○	○
Some unimportant thought runs through my mind and bothers me (17)	○	○	○	○
I take disappointments so keenly that I can't put them out of my mind (18)	○	○	○	○
I am a steady person (19)	○	○	○	○
I get in a state of tension or turmoil as I think over my recent concerns and interests (20)	○	○	○	○

End of Block: STAI

Start of Block: Lumme App Use

lumme_often In a typical week, how often did you use the Lumme app?

- Every day (all 7 days) (1)
- Almost every day (5-6 days) (2)
- Some of the days (3-4 days) (3)
- A few of the days (1-2 days) (4)
- Never (5)

---

lumme_time In a typical day, how much time (in minutes) did you spend on the Lumme app?

____

---

lumme_enjoy Please rank which features of the Lumme app you enjoyed using the most (1) and which you did not enjoy using (16). **Drag and drop the items on the list.**

______ My Profile (1)
______ My Trends (2)
______ My Badges (3)
______ Quit Plan (4)
______ My Diary (5)
______ My Reasons (6)
______ My Supports (7)
______ Instant Help (8)
______ Quit Smoking Tips (9)
______ Quit Plan Reminders (notifications) (10)
______ Motivational Picture (on app home screen) (11)
______ Instant Tip (on app home screen) (12)
______ Money spent/saved (on app home screen) (13)
______ Time since last cigarette (on app home screen) (14)
______ Lumme app widget on home screen (15)
______ Other (please specify): (16)

---

lumme_useful Please rank which features of the Lumme app you found the most useful (1) and which you found the least useful (16) in helping you quit smoking. **<u>Drag and drop the items on the list.</u>**
______ My Profile (1)
______ My Trends (2)
______ My Badges (3)
______ Quit Plan (4)
______ My Diary (5)
______ My Reasons (6)
______ My Supports (7)
______ Instant Help (8)
______ Quit Smoking Tips (9)
______ Quit Plan Reminders (notifications) (10)
______ Motivational Picture (on app home screen) (11)
______ Instant Tip (on app home screen) (12)
______ Money spent/saved (on app home screen) (13)
______ Time since last cigarette (on app home screen) (14)
______ Lumme app widget on home screen (15)
______ Other (please specify): (16)

---

lumme_frequency Please rank which features of the Lumme app you used most frequently (1) and which you used least frequently (16). **<u>Drag and drop the items on the list.</u>**
______ My Profile (1)
______ My Trends (2)
______ My Badges (3)
______ Quit Plan (4)
______ My Diary (5)
______ My Reasons (6)
______ My Supports (7)
______ Instant Help (8)
______ Quit Smoking Tips (9)
______ Quit Plan Reminders (notifications) (10)
______ Motivational Picture (on app home screen) (11)
______ Instant Tip (on app home screen) (12)
______ Money spent/saved (on app home screen) (13)
______ Time since last cigarette (on app home screen) (14)
______ Lumme app widget on home screen (15)
______ Other (please specify): (16)

End of Block: Lumme App Use

Start of Block: Health-ITUES

itues Please select one answer for each question.

	Strongly agree (5)	Somewhat agree (4)	Neither agree nor disagree (3)	Somewhat disagree (2)	Strongly disagree (1)
I think the Lumme App would be a positive addition for persons living with HIV who smoke. (itues1)	◯	◯	◯	◯	◯
I think the Lumme App will improve smoking habits of persons living with HIV. (itues2)	◯	◯	◯	◯	◯
The Lumme App is an important part of helping me quit smoking. (itues3)	◯	◯	◯	◯	◯
Using the Lumme App will make it easier for me to quit smoking. (itues4)	◯	◯	◯	◯	◯
Using the Lumme App enables me to quit smoking more quickly. (itues5)	◯	◯	◯	◯	◯
Using the Lumme App makes it more likely that I can quit smoking. (itues6)	◯	◯	◯	◯	◯

Using the the Lumme App is useful for smoking cessation. (itues7)	○	○	○	○	○
I think the the Lumme App presents a more equitable process for quitting smoking. (itues8)	○	○	○	○	○
I am satisfied with the the Lumme App for smoking cessation. (itues9)	○	○	○	○	○
I am able to quit smoking in a timely manner because of the Lumme App. (itues10)	○	○	○	○	○
Using the Lumme App increases my likelihood of quitting smoking. (itues11)	○	○	○	○	○
I am able to quit smoking when I use the the Lumme App. (itues12)	○	○	○	○	○
I am comfortable with my ability to use the Lumme App. (itues13)	○	○	○	○	○

Learning to operate the Lumme App is easy for me. (itues14)	○	○	○	○	○
It is easy for me to become skillful at using the Lumme App. (itues15)	○	○	○	○	○
I find the Lumme App easy to use. (itues16)	○	○	○	○	○
I can always remember how to log onto and use the Lumme App. (itues17)	○	○	○	○	○
The Lumme App gives error messages that clearly tell me how to fix problems. (itues18)	○	○	○	○	○
When I make a mistake using the Lumme App, I recover easily and quickly. (itues19)	○	○	○	○	○
The information (such as on-line help, on-screen messages, and other documentation) provided with the Lumme App is clear. (itues20)	○	○	○	○	○

uselocation If you have the Lumme App on your smartphone, where would you use it?

- ☐ Home (1)
- ☐ Clinic (2)
- ☐ Library (3)
- ☐ Cafe or restaurant (4)
- ☐ Other (Please specify): (5) ____

End of Block: Health-ITUES

Start of Block: PSSUQ

pssuq Please select one answer for each question.

	Strongly agree (1)	Agree (2)	Somewhat agree (3)	Neither agree nor disagree (4)	Somewhat disagree (5)	Disagree (6)	Strongly disagree (7)
Overall, I am satisfied with how easy it is to use the Lumme app. (pssuq_1)	○	○	○	○	○	○	○
It was simple to use the Lumme app. (pssuq_2)	○	○	○	○	○	○	○
I was able to complete the tasks and scenarios quickly using the Lumme app. (pssuq_4)	○	○	○	○	○	○	○
I felt comfortable using the Lumme app. (pssuq_6)	○	○	○	○	○	○	○
It was easy to learn to use the Lumme app. (pssuq_7)	○	○	○	○	○	○	○
I believe I could become productive quickly using the Lumme app. (pssuq_8)	○	○	○	○	○	○	○
The Lumme app gave error messages that clearly told me how to fix problems. (pssuq_9)	○	○	○	○	○	○	○

Whenever I made a mistake using the Lumme app, I could recover easily and quickly. (pssuq_10)	○	○	○	○	○	○	○
The information (such as on-line help, on-screen messages, and other documentation) provided with the Lumme app was clear. (pssuq_11)	○	○	○	○	○	○	○
It was easy to find the information I needed. (pssuq_12)	○	○	○	○	○	○	○
The information was effective in helping me complete the tasks and scenarios. (pssuq_14)	○	○	○	○	○	○	○
The organization of information on the Lumme app screen was clear. (pssuq_15)	○	○	○	○	○	○	○
The interface of the Lumme app was pleasant. (pssuq_16)	○	○	○	○	○	○	○

I liked using the interface of the Lumme app. (pssuq_17)	○	○	○	○	○	○	○
The Lumme app has all the functions and capabilities I expect it to have. (pssuq_18)	○	○	○	○	○	○	○
Overall, I am satisfied with the Lumme app. (pssuq_19)	○	○	○	○	○	○	○

Page Break

End of Block: PSSUQ

Start of Block: Study Contamination

contamination_1 Do you personally know anyone else who is participating in the Smoking Cessation Pilot study?

○ Yes, someone I know is also participating (1)

○ No, I haven't talked about the study with anyone or the people I have talked about it with are not participating (2)

○ I'm not sure - the study has been mentioned but I don't know if anyone I've talked to is participating (3)

Display This Question:

If contamination_1 != 2

And arm = 4

X→

contamination_2 Did you talk about the app or smoking strategies with another person participating in the study?

- Yes (1)
- No (2)
- I'm not sure (3)

Page Break

X→

contamination_3 Which Smoking Cessation Pilot study group do you think you're in?

- I'm pretty sure I'm in the intervention group (1)
- I think I'm in the intervention group (2)
- I don't know (3)
- I think I'm in the control group (4)
- I'm pretty sure I'm in the control group (5)

Page Break

Display This Question:

If contamination_3 = 1

Or contamination_3 = 2

contamination_4 Why do you think you're in the intervention group?

____

Display This Question:

If contamination_3 = 4

Or contamination_3 = 5

contamination_5 Why do you think you're in the control group?

____

End of Block: Study Contamination

Start of Block: COVID-19 v2

covid_dx_v2 In the last 3 months (since ${e://Field/covid_todayminus90}), have you been diagnosed with COVID-19 by a doctor or another health care provider?

- Yes (1)
- No (2)
- I don't know (999)
- I was diagnosed with COVID-19 prior to ${e://Field/covid_todayminus90} (888)

Display This Question:

If covid_dx_v2 = 1

Or covid_dx_v2 = 888

covid_hosp_v2 Were you hospitalized due to your COVID-19 diagnosis?

- Yes (1)
- No (2)

covid_dx_others_v2 In the last 3 months (since ${e://Field/covid_todayminus90}), have any of your close friends or family members been diagnosed with COVID-19 by a doctor or another health care provider?

- Yes (1)
- No (2)
- I don't know (999)

---

covid_concern_v2 Overall, how concerned are you about the COVID-19 pandemic?

- Not at all concerned (0)
- A little concerned (1)
- Somewhat concerned (2)
- Very concerned (3)

Page Break

covid_order_v2 Due to the COVID-19 pandemic, several cities, counties, and states have had one or more “Shelter in Place/Stay at Home” orders or advisories. Additionally, some people have voluntarily sheltered in place/stayed at home for different periods of time. By this we mean that you stayed at home and only left home to get food, medicine, heath care services, or for work if you are an essential worker. In the last 3 months (since ${e://Field/covid_todayminus90}), have you sheltered-in-place/stayed-at-home either voluntarily or due to a city, county, or state mandate/advisory due to the COVID-19 pandemic?

- Yes (1)
- No (2)

Skip To: covid_medcare_v2 If covid_order_v2 = 2

---

covid_order_desc_v2 Which of the following best describes your voluntary or mandated shelter-in-place/stay-at-home in the last 3 months, since ${e://Field/covid_todayminus90}?

◯ Have continued to "Shelter in Place/Stay at Home" and have been since before ${e://Field/covid_todayminus90} (1)

◯ Stopped a “Shelter in Place/Stay at Home” that started prior to ${e://Field/covid_todayminus90} and have not been sheltering/staying at home since (2)

◯ Stopped a “Shelter in Place/Stay at Home” that started prior to ${e://Field/covid_todayminus90} and have since engaged in another “Shelter in Place/Stay at Home” (3)

◯ Have only started a “Shelter in Place/Stay at Home” (4)

Display This Question:

If covid_order_desc_v2 = 2

Or covid_order_desc_v2 = 3

covid_order_stop_v2 You said you stopped a “Shelter in Place/Stay at Home” that started prior to ${e://Field/covid_todayminus90}. On approximately, what date did you stop?

◯ End Date (MM/DD/YYYY) (1) ____

Display This Question:

If covid_order_desc_v2 = 3

Or covid_order_desc_v2 = 4

covid_order_type_v2 You said you have begun a “Shelter in Place/Stay at Home” in the last 3 months due to the COVID-19 pandemic. Is this a city, county, state or other locale mandated order/advisory, or are you voluntarily sheltering in place?

◯ City, County, State or Locale Mandated/Advisory “Shelter in Place/Stay at Home” (1)

◯ Voluntary “Shelter in Place/Stay at Home” (2)

Display This Question:

If covid_order_desc_v2 = 3

Or covid_order_desc_v2 = 4

And If

covid_order_type_v2 = 1

covid_order_fllw_v2 Which government-level **Shelter in Place/Stay at Home Orders** are you following? *(Please select all that apply.)*

- ▢ City (please specify) (1) ____
- ▢ County (please specify) (2) ____
- ▢ State (please specify) (3) ____

Display This Question:

If covid_order_desc_v2 = 3

Or covid_order_desc_v2 = 4

quar_actv_v2 Are you still sheltering in place/staying at home due to COVID-19?

- ○ Yes (1)
- ○ No (2)

Display This Question:

If covid_order_desc_v2 = 3

Or covid_order_desc_v2 = 4

quar_dates_v2 On approximately what date did your “**Shelter in Place/Stay at Home**” start (and end, if applicable) in the last 3 months? (Example: 05/14/2020)

○ Start Date (MM/DD/YYYY) (1) ____

Display This Choice:
If quar_actv_v2 = 2

○ End Date (MM/DD/YYYY) (2) ____

X→

covid_medcare_v2 In the last 3 months (since ${e://Field/covid_todayminus90}), how concerned have you been about your ability to access medical care during the COVID-19 outbreak?

○ Not at all concerned (0)

○ A little concerned (1)

○ Somewhat concerned (2)

○ Very concerned (3)

X→

art_covid_v2 In the last 3 months (since ${e://Field/covid_todayminus90}), have you used ART (HIV medication)?

○ Yes (1)

○ No (0)

Skip To: covid_art_conc_v2 If art_covid_v2 = 0

X→

covid_art_change_v2 In the last 3 months (since ${e://Field/covid_todayminus90}) during the COVID-19 outbreak, have you changed how you take ART (HIV medication)?

○ No, my ART use hasn't changed (0)

○ Yes, I stopped taking ART as often as I did before (1)

○ Yes, my ART use changed in some ways but did not stop completely (please specify) (2) ____

Skip To: art_prov_contact_v2 If covid_art_change_v2 = 0

covid_stop_art_v2 Why did you stop taking ART (HIV medication) as often or completely?

○ I don't think I need it anymore (please specify) (1) ____

○ I don't have access to it anymore (2)

○ I stopped for some other reason (please specify) (3) ____

Skip To: End of Block If covid_stop_art_v2 = 1

Display This Question:

If covid_stop_art_v2 = 2

covid_art_noacc_v2 Why do you not have access to ART (HIV medication) anymore?

○ I don't have health insurance anymore (1)

○ My provider can't prescribe or refill my prescription (2)

○ I can't complete routine testing/labs required for my prescription (3)

○ Some other reason (please specify) (4) ____

art_prov_contact_v2 In the last 3 months (since ${e://Field/covid_todayminus90}), have you reached out to your provider for an ART (HIV medication) refill?

◯ Yes, I have (1)

◯ No, I have not (0)

◯ No, I have more than a 90-day supply (999)

Display This Question:

If art_prov_contact_v2 = 1

X→

covid_prov_offer_v2 Did your provider offer any of the following services when you reached out about an ART (HIV medication) refill?

◯ My provider offered a refill without the quarterly visit (1)

◯ My provider offered a telemedicine (e.g., video call) appointment to refill my prescription (2)

◯ My provider offered an office-based appointment to refill my prescription (3)

◯ My provider offered something else (please specify) (4) ____

X→

covid_art_conc_v2 In the last 3 months (since ${e://Field/covid_todayminus90}), how concerned have you been about accessing ART (HIV medication) during the COVID-19 outbreak?

◯ Not at all concerned (0)

◯ A little concerned (1)

◯ Somewhat concerned (2)

◯ Very concerned (3)

End of Block: COVID-19 v2

Start of Block: Last Question

Display This Question:

If Visit_Type = 2

Q69

Thank you for completing your 12-week Follow Up Survey for the Smoking Pilot! Please contact a staff member at 212-305-8198 to let them know you have completed the survey.

End of Block: Last Question
